# Supplementary material for: A simple mechanism for integration of quorum sensing and cAMP signalling in V. cholerae
Source: bioRxiv. 2023 May 26:2023.02.08.527633. Originally published 2023 Feb 8. Preprint. [Version 2] doi: 10.1101/2023.02.08.527633 (PMC9934648; doi:10.1101/2023.02.08.527633)
Supplement: Supplement 1 [file media-1.pdf]

Table S1: HapR binding targets identified by previous studies

gene(s)	evidence[1]	source	detected by ChIP-seq[2]
VC0583	EMSA, DNAse I footprint	(1)	yes
VC2647	EMSA, DNAse I footprint	(2)	no
VCA0865	not shown directly	(3)	yes (below cut-off)
VCA0952	EMSA	(4)	yes (below cut-off)
VC2370	EMSA	(4)	no
VC0900	EMSA	(4)	no
VC1851	EMSA	(4)	yes
VC1086	EMSA	(4)	no
VCA0074	EMSA	(4)	no
VCA0246<>VCA0247	EMSA	(5)	yes (below cut-off)
VC1222	EMSA	(5)	no
VC1181	EMSA	(5)	no
VC0432	EMSA	(5)	no
VC2762	EMSA	(5)	no
VC1000	EMSA	(5)	no
VC2634<>VC2635	EMSA	(5)	no
VCA0017	EMSA	(5)	yes (below cut-off)
VCA0182<>VCA0183	EMSA	(5)	no
VC0166<>VC0167	EMSA	(5)	no
VC1415	EMSA	(5)	yes (below cut-off)
VCA0880	EMSA	(5)	yes (below cut-off)
VCA0684	EMSA	(5)	no
VC2674	EMSA	(5)	no
VC1213	EMSA	(5)	yes (below cut-off)
VC2035	EMSA	(5)	no
VC0089	EMSA	(5)	no
VCA0865	EMSA	(5)	yes (below cut-off)
VCA0148	EMSA	(5)	yes
VC0934	EMSA	(5)	no
VC0241	EMSA	(5)	yes

[1]The column lists evidence for direct binding either using electrophoretic mobility shift assay (EMSA) or DNAse I footprinting.

[2]The column lists evidence for binding from ChIP-seq assays. In cases where a binding peak was evident upon visual inspection, but this fell below our stringent cut-off, this is indicated.

1. Lin, W., Kovacikova, G. & Skorupski, K. Requirements for Vibrio cholerae HapR binding and transcriptional repression at the hapR promoter are distinct from those at the aphA promoter. *J. Bacteriol.* **187**, 3013–3019 (2005).

2. Kovacikova, G. & Skorupski, K. Regulation of virulence gene expression in Vibrio cholerae by quorum sensing: HapR functions at the aphA promoter. *Mol. Microbiol.* **46**, 1135–1147 (2002).

3. Silva, A. J., Pham, K. & Benitez, J. A. Haemagglutinin/protease expression and mucin gel penetration in El Tor biotype Vibrio cholerae. *Microbiology* **149**, 1883–1891 (2003).

4. Waters, C. M., Lu, W., Rabinowitz, J. D. & Bassler, B. L. Quorum sensing controls biofilm formation in Vibrio cholerae through modulation of cyclic Di-GMP levels and repression of vpsT. *J. Bacteriol.* **190**, 2527–2536 (2008).

5. Tsou, A. M., Cai, T., Liu, Z., Zhu, J. & Kulkarni, R. V. Regulatory targets of quorum sensing in Vibrio cholerae: Evidence for two distinct HapR-binding motifs. *Nucleic Acids Res.* **37**, 2747–2756 (2009).
