# Supplementary material for: A simple mechanism for integration of quorum sensing and cAMP signalling in V. cholerae
Source: bioRxiv. 2023 May 26:2023.02.08.527633. Originally published 2023 Feb 8. Preprint. [Version 2] doi: 10.1101/2023.02.08.527633 (PMC9934648; doi:10.1101/2023.02.08.527633)
Supplement: Supplement 2 [file media-2.pdf]

Table S2: Strains, plasmids, and oligonucleotides

Name	Description	Source
	**Bacterial Strains**	
	Vibrio cholerae[1]	
E7946	Wildtype *Vibrio cholerae* El Tor Ogawa derivative, SmR	(1)
E7946 Δ*hapR*	E7946 derivative lacking *hapR*	This work
E7946 Δ*crp*	E7946 derivative lacking *crp*	This work
E7946 Δ*hapR*Δ*crp*	E7946 derivative lacking *hapR* and *crp*	This work
E7946 Δ*luxO*	E7946 derivative lacking *luxO*	This work
E7946 Δ*hapR*	E7946 derivative *hapR::Spec^R*	This work
E7946 Δ*luxO*Δ*hapR*	E7946 derivative lacking *luxO*, *hapR::Spec^R*	This work
E7946 Δ*murP*	E7946 derivative *lacking murP*	(2)
	Escherichia coli	
DH5α	*fhuA*2 Δ(*argF*-*lacZ*)U169 *phoA glnV*44 Φ80 Δ(*lacZ*)M15 *gyrA*96 *recA*1 *relA*1 *endA*1 *thi*-1 *hsdR*17	NEB
T7 Express	*fhuA2 lacZ::T7 gene1 [lon] ompT gal sulA11 R(mcr-73::mini Tn10*--Tet^S*)2 [dcm] R(zgb-210::Tn10*--Tet^S*) endA1 Δ(mcrC-mrr)114::IS10*	NEB
JCB387	Δ*nirB*, Δ*lac*	(3)
S17 λpir	*lac*U169 (*lac*ZM15), *recA*1, *endA*1, *hsdR*17, *thi*-1, *gyrA*96, *relA*1, *pir*	(4)
	**Plasmids**	
pSR	pBR322-derived plasmid. Features cloning site upstream λoop transcription terminator. AmpR	(5)
pRW50T	A broad-host range *lacZ* expression vector encoding *oriT* of pRK, TetR, Tra+	(6)
pAMNF	Plasmid for basal expression of N-terminal 3xFLAG-tagged proteins, KanR	(7)
pAMCF	Plasmid for basal expression of C-terminal 3xFLAG-tagged proteins, KanR	(7)
pRK2013	Helper plasmid for conjugation, KanR, oriColE1, RK2-, Mob+, Tra+	(8)
pKAS32	Suicide plasmid for mutant strain construction, AmpR	(9)
	**Oligonucleotides[2,3]**	
	Oligonucleotides for cloning *hapR* or *luxO* (5' to 3')	
hapR|pAMNF|fwd	GGCTGC*GGTACC*ATGGACGCATCAATCGAAAAACGC	This work
hapR|pAMNF|rev	GCCCG*AAGCTT*CTAGTTCTTATAGATACACAGCATATTGAGG	This work

luxO|pAMCF|fwd	GGCTGC*GGTACC*ATGGTAGAAGACACGGCGTCGGTG GCGGCGCTGTATCGTTCTTACCTCACACCGCTGGATA TTGATATCAATATCGTGGGTACGGGAC	This work
luxO|pAMCF|rev.1	ATCGCGTCCCGTACCCACGATATTG	This work
luxO|pAMCF|rev.2	GCCCG*AAGCTT*CCGTTCCTTCTCTTTTTCTTTCAC	This work

Oligonucleotides for cloning regulatory regions in pRW50T and pSR (5' to 3')

P*murQ*|fwd	AAAA*GAATTC*CACCAATCTGGCGGCCACTC	This work
P*murQ*|rev	TTTT*AAGCTT*CATAAGGCTTCTCGGCAAAT	This work
P*hapR*|fwd	AAAA*GAATTC*CATACCATTCTCGTTGTGTT	This work
P*hapR*|rev	TTTT*AAGCTT*CATAGGGGTATATCCTTGCC	This work
P*VC0585*|fwd	AAAA*GAATTC*CATAGGGGTATATCCTTGCC	This work
P*VC0585*|rev	TTTT*AAGCTT*CATACCATTCTCGTTGTGTT	This work
P*VC0502*|fwd	AAAA*GAATTC*GACAAAAGTTTGTTGCCCGC	This work
P*VC0502*|rev	TTTT*AAGCTT*CATAGTATTGGCTTTGGCAT	This work
P*mutH*|fwd	AAAA*GAATTC*ACCAGACCACATGAAGATCC	This work
P*mutH*|rev	TTTT*AAGCTT*CATAAAGGCTTTCGGTTTGG	This work
P*leuO*|fwd	AAAA*GAATTC*TAATGAACTGACTAACTCA	This work
P*leuO*|rev	TTTT*AAGCTT*CATTGCGTCTTTTTTATCT	This work
P*VC0433*|fwd	AAAA*CAATTG*TACCTGCAACTTCAAGTAGT	This work
P*VC0433*|rev	TTTT*AAGCTT*CATTATTTTTTAATCACCAA	This work
P*VC0241*|fwd	AAAA*GAATTC*CTATAGATGCGAACAGTTGC	This work
P*VC0241*|rev	TTTT*AAGCTT*CATAGTATGCTGACTACTGC	This work
P*rfaD*|fwd	AAAA*CAATTG*CATAGTATGCTGACTACTGC	This work
P*rfaD*|rev	TTTT*AAGCTTC*ATTATGAATTCCTATAGAT	This work
P*VC0620*|fwd	AAAA*GAATTC*AAACCGTACCCGTTTTGCGAG	This work
P*VC0620*|rev	TTTT*AAGCTT*ACTAGTAAGGAACAGCTATG	This work
P*VC0688*|fwd	AAAA*GAATTC*CATAGCGTTTGTCCTTTTGT	This work
P*VC0688*|rev	TTTT*AAGCTTC*ATTACTATCCATTTTTTCA	This work
P*VC1298*|fwd	AAAA*GAATTC*CATAAACTCTTTGTATAATT	This work
P*VC1298*|rev	TTTT*AAGCTT*CATGATAGTTTTGTAATTAT	This work
P*VC1375*|fwd	AAAA*CAATTG*CATCCACTTCTTCCTTATTA	This work
P*VC1375*|rev	TTTT*AAGCTT*CATATCAAAAGTGGTTGGGA	This work
P*VC1376*|fwd	AAAA*CAATTG*CATATCAAAAGTGGTTGGGA	This work
P*VC1376*|rev	TTTTAAGCTTCATCCACTTCTTCCTTATTA	This work
P*VC1403*|fwd	AAAA*GAATTC*CATTTTTTGGGTAATCGATA	This work
P*VC1403*|rev	TTTT*GGATCC*CATGGAAAACCTCGTTGTTT	This work
P*VC1405*|fwd	AAAA*GAATTC*CATGGAAAACCTCGTTGTTT	This work

P*VC1405*|rev	TTTT*GGATCC*CATTTTTTGGGTAATCGATA	This work
P*VC1436*|fwd	AAAA*GAATTC*CCAATTCGTAAAGAAGCTAA	This work
P*VC1436*|rev	TTTT*GGATCC*CATATCTGCTGAGGCTTTAG	This work
P*VC2352*|fwd	AAAA*GAATTC*TCAGCACAATATCTCGCGCC	This work
P*VC2352*|rev	TTTT*AAGCTT*CATGCCGATGAGGCTCATAA	This work
P*VCA0218*|fwd	AAAA*GAATTC*CATATAAACCTCACTGACTC	This work
P*VCA0218*|rev	TTTT*AAGCTT*CATCGTTTACTTCTTATCAT	This work
P*VCA0219*|fwd	AAAA*GAATTC*CATCGTTTACTTCTTATCAT	This work
P*VCA0219*|rev	TTTT*AAGCTT*CATATAAACCTCACTGACTC	This work
P*VCA0662*|fwd	AAAA*GAATTC*TCAGTTTGTAAATTGAGGAA	This work
P*VCA0662*|rev	TTTT*AAGCTT*CATATCAAATATGAATCCTT	This work
P*VCA0663*|fwd	AAAA*GAATTC*CATATCAAATATGAATCCTT	This work
P*VCA0663*|rev	TTTT*AAGCTT*CATGGTGTTACCTACTTGTT	This work
P*VCA0906*|fwd	AAAA*GAATTC*ATTTTTATCCTGACCCCATA	This work
P*VCA0906*|rev	TTTT*GGATCC*CATGCCTACGCCTATCGCCG	This work
P*VCA0960*|fwd	AAAA*GAATTC*CATCATGAGTTATATTTACA	This work
P*VCA0960*|rev	TTTT*AAGCTT*CATCCCTCAATCCTCAGTTT	This work
P*VCA0961*|fwd	AAAA*GAATTC*CATCCCTCAATCCTCAGTTT	This work
P*VCA0961*|rev	TTTT*AAGCTT*CATCATGAGTTATATTTACA	This work

Oligonucleotides for sequencing cloned DNA in plasmid constructs (5' to 3')

pRW50|seq|fwd	GTTCTCGCAAGGACGAGAATTTC	This work
pRW50|seq|rev	GTCGTTGAACTGAGCCTGAAATTCAGG	This work
pSR|seq|fwd	GTGCCACCTGACGTCTAAGAAACC	This work
pSR|seq|rev	GCAACCGAGCGTTCTGAACAAATCC	This work
pAM|seq|fwd	ATTTATTCCAATGTCACACACTTTTCGC	This work
pAM|seq|rev	GAAACGCCGTAGCGCCGATGGTAGT	This work

Oligonucleotides for amplifying DNA for radio labelling (5' to 3')

pSR|footprinting|fwd	[BIOTIN]-CACGAGGCCCTTTCGTCTTCTC	This work
pSR|footprinting|rev	GGAGTTCTGAGGTCATTACTGGAG	This work
pSR|footprinting|rev	CACGAGGCCCTTTCGTCTTCTC	This work
pSR|footprinting|rev	[BIOTIN]-GGAGTTCTGAGGTCATTACTGGAG	This work

Oligonucleotides for cloning DNA fragments in pKAS32 (5' to 3')

pKAS32|fwd	GCAGGCACAAGCGGCCGCCTGCAGCTGGCGCCA TCGATACGCGTACGTCG	This work
pKAS32|rev	CACGGTTTCATTAACAACCGGTACCTCTAGAACT	This work

	ATAGCTAGCATGCGCAAATTTAAAGCGCTG	This work
crp|arm|up|fwd	CGGTTGTTAATGAAACCGTGGATATTAAATGC	This work
crp|arm|up|rev	ATCGGGGCACCTAGCCGATTTTTCCGGTTTC	This work
crp|arm|down|fwd	AATCGGCTAGGTGCCCCGATAACCCGTC	This work
crp|arm|down|rev	AGGCGGCCGCTTGTGCCTGCGCAGCCAA	This work
hapR|arm1|fwd	CTAGAGGTACCGGTTGTTAATCCCAACCCCGATT GGTAATC	This work
hapR|arm1|rev	TGTGTTTCATTTTCTTGGGCAGCACAAAG	This work
hapR|arm2|fwd	GCCCAAGAAAATGAAACACACAGTTGAAGTC	This work
hapR|arm2|rev	CGCCAGCTGCAGGCGGCCGCTATGCGGTCGATGT GCTGA	This work
	Oligonucleotides for mutagenesis of *crp* or *hapR* (5' to 3')	
hapR|R123E|fwd	TGCTTCAACCGAGGACGAAGTTTGGC	This work
hapR|R123A|fwd	TGCTTCAACCGCTGACGAAGTTTGGCC	This work
hapR|R123|rev	CTCCACTCAAACCAGACTTTG	This work
crp|E55R|fwd	GATCAAAGATCGTGAAGGTAAAGAGATGATTCTC	This work
cpr|E55A|fwd	GATCAAAGATGCGGAAGGTAAAGAGATGATTC	This work
crp|E55|rev	AGTACCGCAACTGAACCT	This work

[1]E7946 Δ*luxO*, Δ*hapR*, Δ*lux*OΔ*hapR* and Δ*murP* derivatives are synonymous with strains SAD764, SAD793, TND3765 and SAD268.

[2]sequences in italic are sites for restriction endonucleases for use during cloning

[3]underlined sequences encode amino acid substitutions or remove unwanted sites for restriction endonucleases

REFERENCES

1. Levine,M.M., Black,R.E., Clements,M.L., Cisneros,L., Saah,A., Nalin,D.R., Gill,D.M., Craig,J.P., Young,C.R. and Ristaino,P. (1982) The pathogenicity of nonenterotoxigenic vibrio cholerae serogroup 01 biotype ei tor isolated from sewage water in brazil. *J. Infect. Dis.*, **145**, 296–299.

2. Hayes,C.A., Dalia,T.N. and Dalia,A.B. (2017) Systematic genetic dissection of PTS in Vibrio cholerae uncovers a novel glucose transporter and a limited role for PTS during infection of a mammalian host. *Mol. Microbiol.*, **104**, 568–579.

3. Page,L., Griffiths,L. and Cole,J.A. (1990) Different physiological roles of two independent pathways for nitrite reduction to ammonia by enteric bacteria. *Arch. Microbiol.*, **154**, 349–354.

4. De Lorenzo,V., Cases,I., Herrero,M. and Timmis,K.N. (1993) Early and late responses of TOL promoters to pathway inducers: identification of postexponential promoters in Pseudomonas putida with lacZ-tet bicistronic reporters. *J. Bacteriol.*, **175**, 6902.

5. Kolb,A., Kotlarz,D., Kusano,S. and Ishihama,A. (1995) Selectivity of the Escherichia coli RNA polymerase Eσ38 for overlapping promoters and ability to support CRP activation. *Nucleic Acids Res.*, **23**, 819–826.

6. Manneh-Roussel,J., Haycocks,J.R.J., Magán,A., Perez-Soto,N., Voelz,K., Camilli,A., Krachler,A.M. and Grainger,D.C. (2018) Camp receptor protein controls Vibrio cholerae gene expression in response to host colonization. *MBio*, **9**.

7. Haycocks,J.R.J., Sharma,P., Stringer,A.M., Wade,J.T. and Grainger,D.C. (2015) The Molecular Basis for Control of ETEC Enterotoxin Expression in Response to Environment and Host. *PLoS Pathog.*, **11**, 1–16.

8. Ditta,G., Stanfield,S., Corbin,D., Helinski,D.R. and Donald Helinskl,C.R. (1980) Broad host range DNA cloning system for Gram-negative bacteria: Construction of a gene bank of Rhizobium meliloti (plasmid RK2/plasmid vehicle/conjugal transfer/nif genes). *Genet. Downloaded Univ. Birmingham*, **77**, 7347–735.

9. Skorupski,K. and Taylor,R.K. (1996) Positive selection vectors for allelic exchange. *Gene*, **169**, 47–52.
