# Supplementary material for: A simple mechanism for integration of quorum sensing and cAMP signalling in V. cholerae
Source: bioRxiv. 2023 May 26:2023.02.08.527633. Originally published 2023 Feb 8. Preprint. [Version 2] doi: 10.1101/2023.02.08.527633 (PMC9934648; doi:10.1101/2023.02.08.527633)
Supplement: Supplement 3 — Figure 1-figure supplement 1: Binding of LuxO and the qrr1 and VC1142 loci. a. ChIP-seq coverage plots for individual experimental replicates. Signals above or below the horizontal line correspond to reads mapping to the top or bottom strand respectively. b. Sequence of the intergenic region between VC1142 and VC1143. The LuxO target site is shown in red. Start codons for the divergent genes VC1142 and VC1143 are in green. Transcription start sites identified by Papenfort et al32 are in uppercase with arrows indicating the direction of transcription. Underlined sequences are potential promoter −10 elements for the housekeeping RNA polymerase assuming a minimal requirement for the sequence 5'-TANNNT-3'. [file media-3.pdf]

Figure 1 figure supplement 1

a

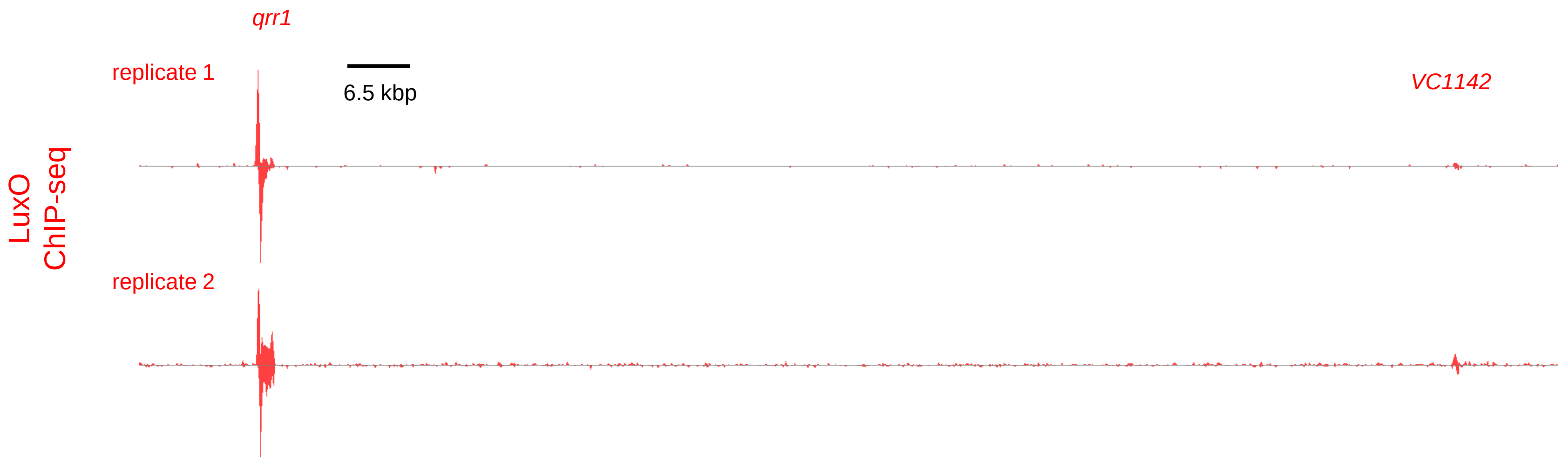

b

tata**cat**ccctc**A**tgcattttataactgatgttttaagagaaacgtcaatttgtccctaggacttcctgacttctcaaaagtgaata
gcgattcagcctatgcttaacgttagtcac**T**aattgacc**aattta**gacgatgattgaacgcagtcaataggacgaacgtataaaagc
acacagtttgatatgcaaactcaattttg**C**aattgca**attata**taaatttgtcat**ttgcaaatcgcga**agcaaatcattaacaagct
tgcatattggtgtcggtaaagtaagcaatcgcgctattcgtgatcgtcattcgcaatgttttatttgcagcgt**tacaat**tgctgc**A**t
tagtctctaagt**A**agaggcgaaattcattaaagtgcggatgccgtcagtgagtttaaacggctagttttacagcactttatagttga
attctctgtaagaagccctcaaatattgaagt**G**tc**atg**
