# Supplementary figures and images for: A simple mechanism for integration of quorum sensing and cAMP signalling in V. cholerae
Source: bioRxiv. 2023 May 26:2023.02.08.527633. Originally published 2023 Feb 8. Preprint. [Version 2] doi: 10.1101/2023.02.08.527633 (PMC9934648; doi:10.1101/2023.02.08.527633)

Figure 1-figure supplement 2

a

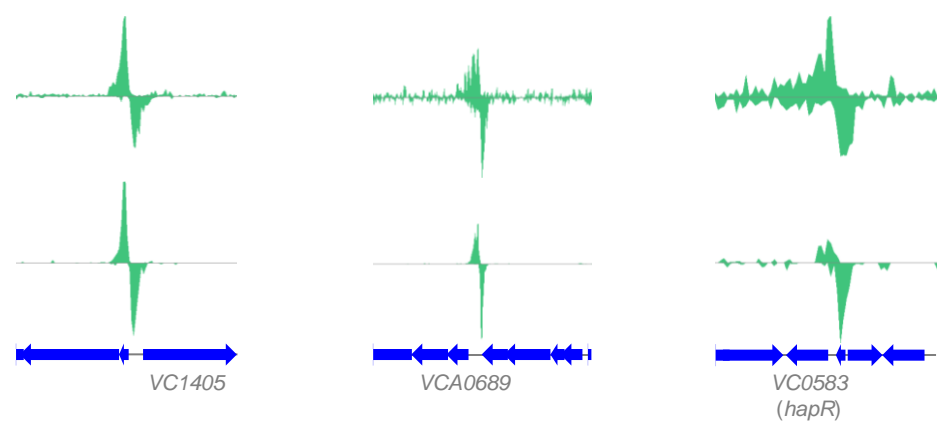

b

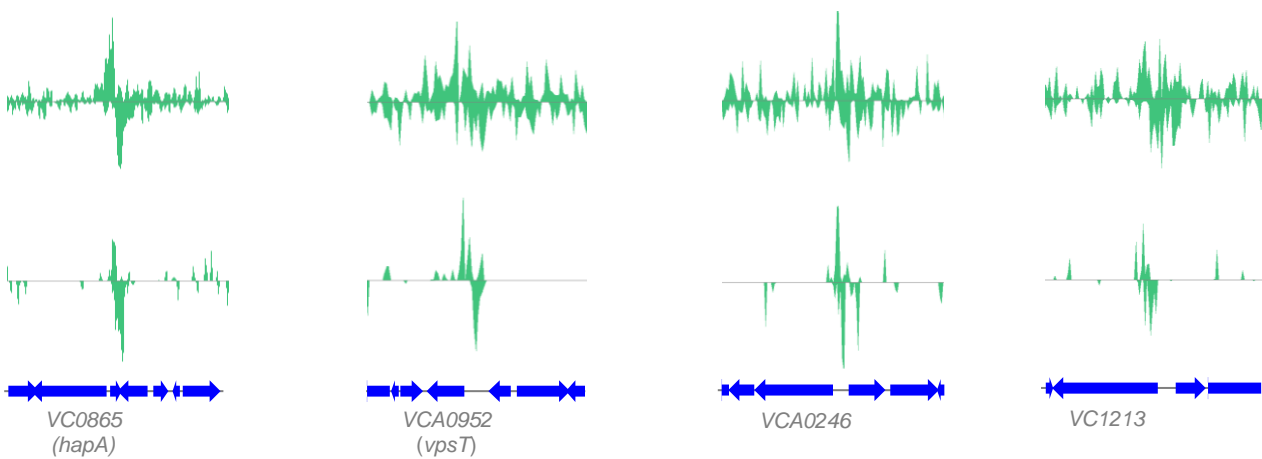

Supplement: Supplement 4 — Figure 1-figure supplement 2: Example HapR binding signals. a. Binding peaks for HapR that fall above our cut-off for peak selection. The HapR ChIP-seq binding profiles are shown in green and genes are shown as blue arrows. b. Binding peaks for HapR, at known targets, that fall below our cut-off for peak selection. Binding signals for HapR are shown at known target genes. These peaks for not selected by our analysis because the signal was too weak and/or insufficiently reproducible. [file media-4.pdf]

Figure 4 figure supplement 1

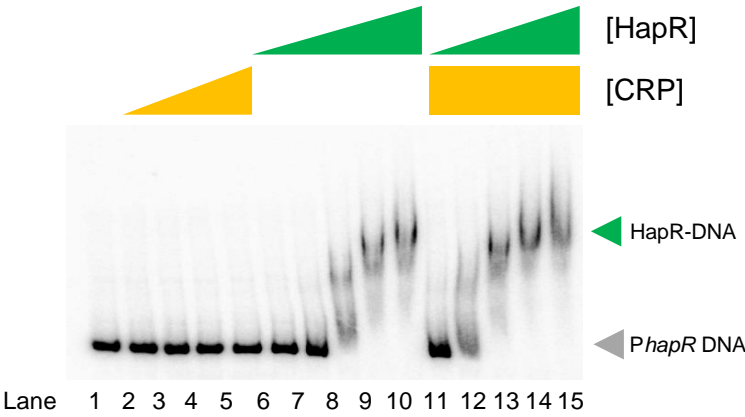

Supplement: Supplement 5 — Figure 4-figure supplement 1: Binding of HapR to the hapR promoter region in the presence and absence of CRP. Electrophoretic mobility shift assay showing migration of the hapR regulatory region with different combinations of CRP (0.0125, 0.025, 0.05 or 0.1 μM) and HapR (0.0625, 0.125, 0.25 or 0.5 μM). For incubations with both factors, the same range of HapR concentrations was used with 0.1 μM CRP. [file media-5.pdf]

Figure 5-figure supplement 1

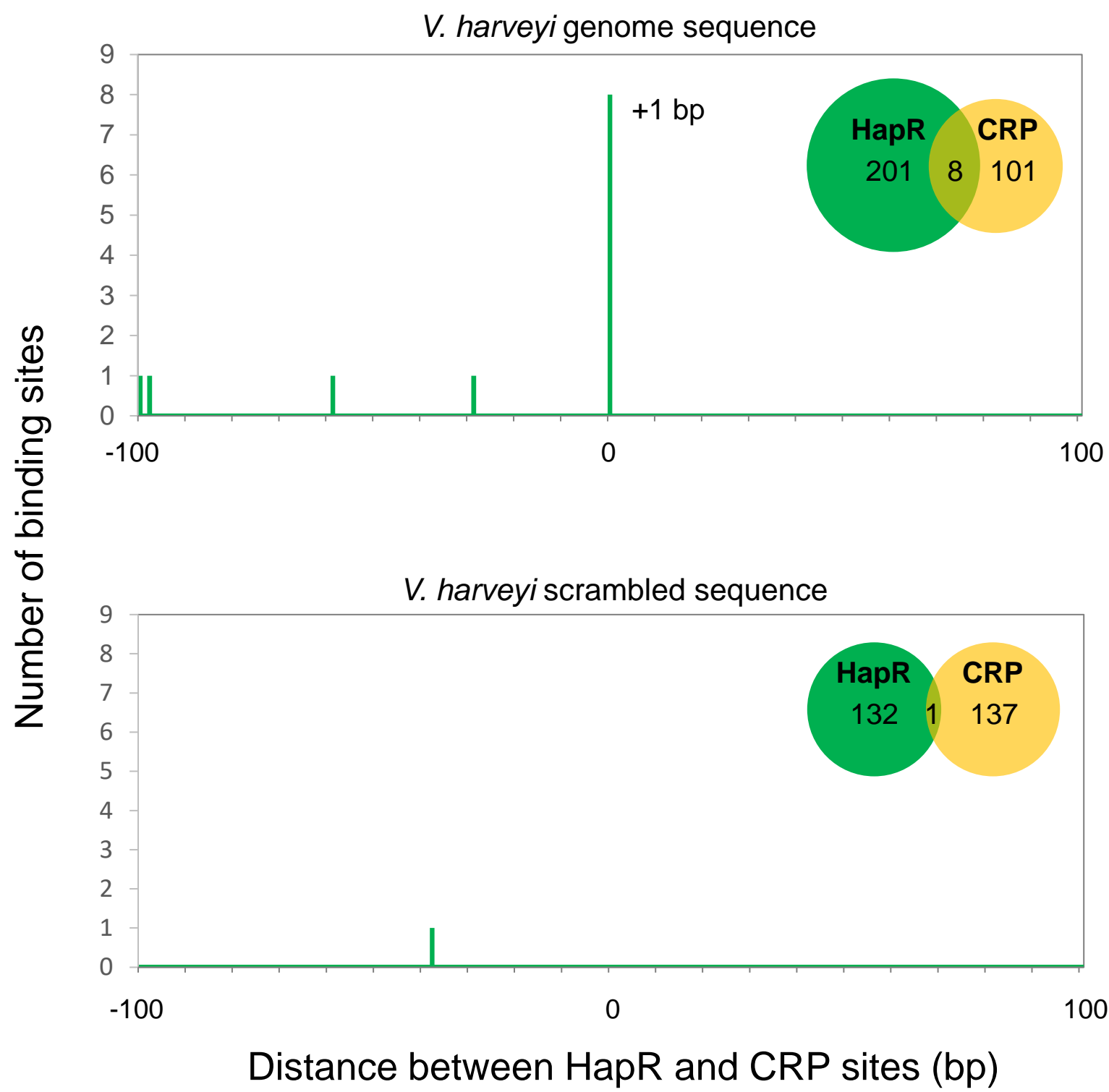

Supplement: Supplement 6 — Figure 5-figure supplement 1: Global overlap of CRP and HapR binding sites in Vibrio harveyi. A position weight matrix (PWM), corresponding to each DNA sequence logo shown in Figure 5a, was created. The PWMs were used to search the V. harveyi genome sequence (strain ATCC 33843) using FIMO. Distances between the identified CRP and HapR sites were calculated. Proximal sites were always overlapping and offset by one base pair (top panel). Overlap was greatly reduced when the analysis was applied to a randomised version of the same genome sequence (bottom panel). [file media-6.pdf]

Figure 5-figure supplement 2

a

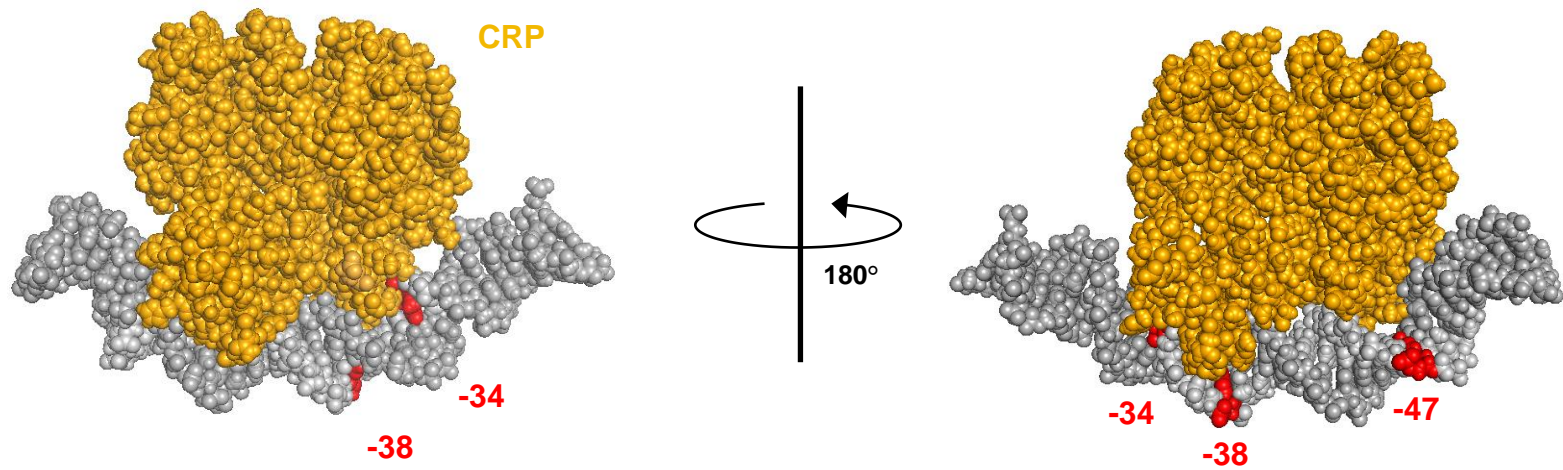

DNase I hypersensitivity

b

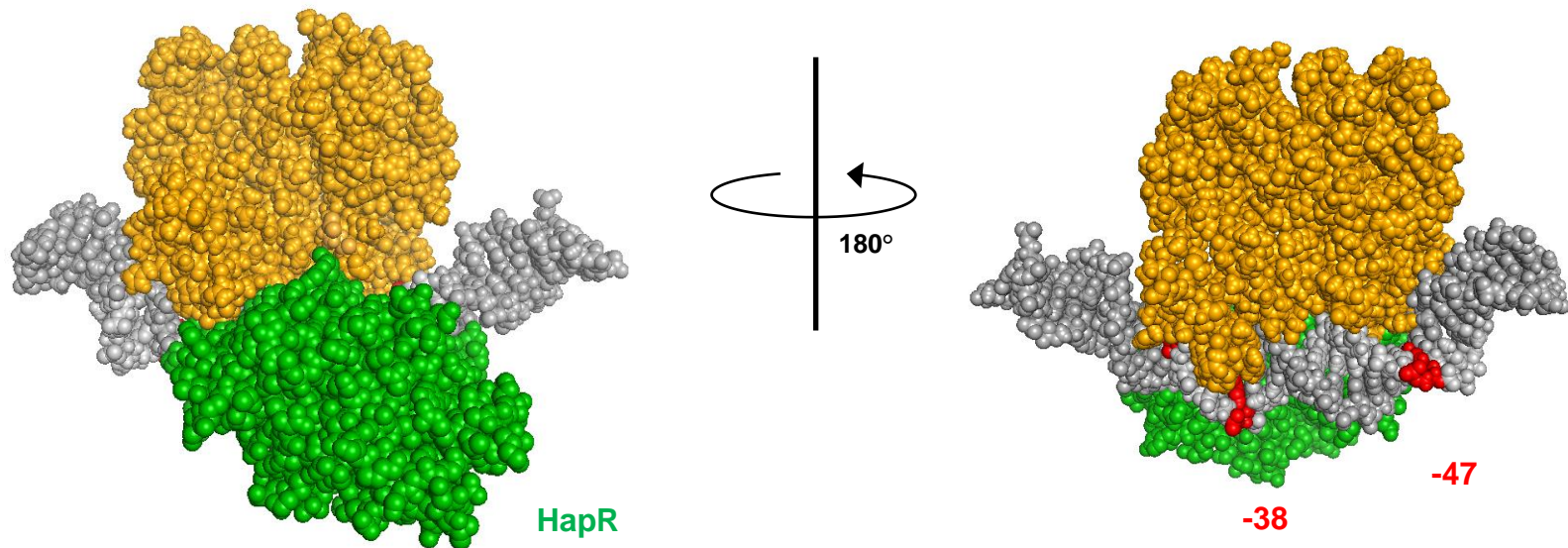

Supplement: Supplement 7 — Figure 5-figure supplement 2: Models of the DNA-CRP and DNA-CRP-HapR complexes. The models were generated using PDB submissions 6pb6 (E. coli CRP in complex with a class II CRP dependent promoter) and 1jt0 (S. aureus QacR bound to its DNA target). The DNA is shown in grey and positions hypersensitive to DNAse I cleave, in the context of the DNA-CRP complex, are highlighted red (Figure 4a). DNA position −34 is not cleaved by DNAse I in the context of the ternary DNA-CRP-HapR complex (Figure 4a). Consistent with this, position −34 is obscured by HapR binding. [file media-7.pdf]

Figure 5-figure supplement 3

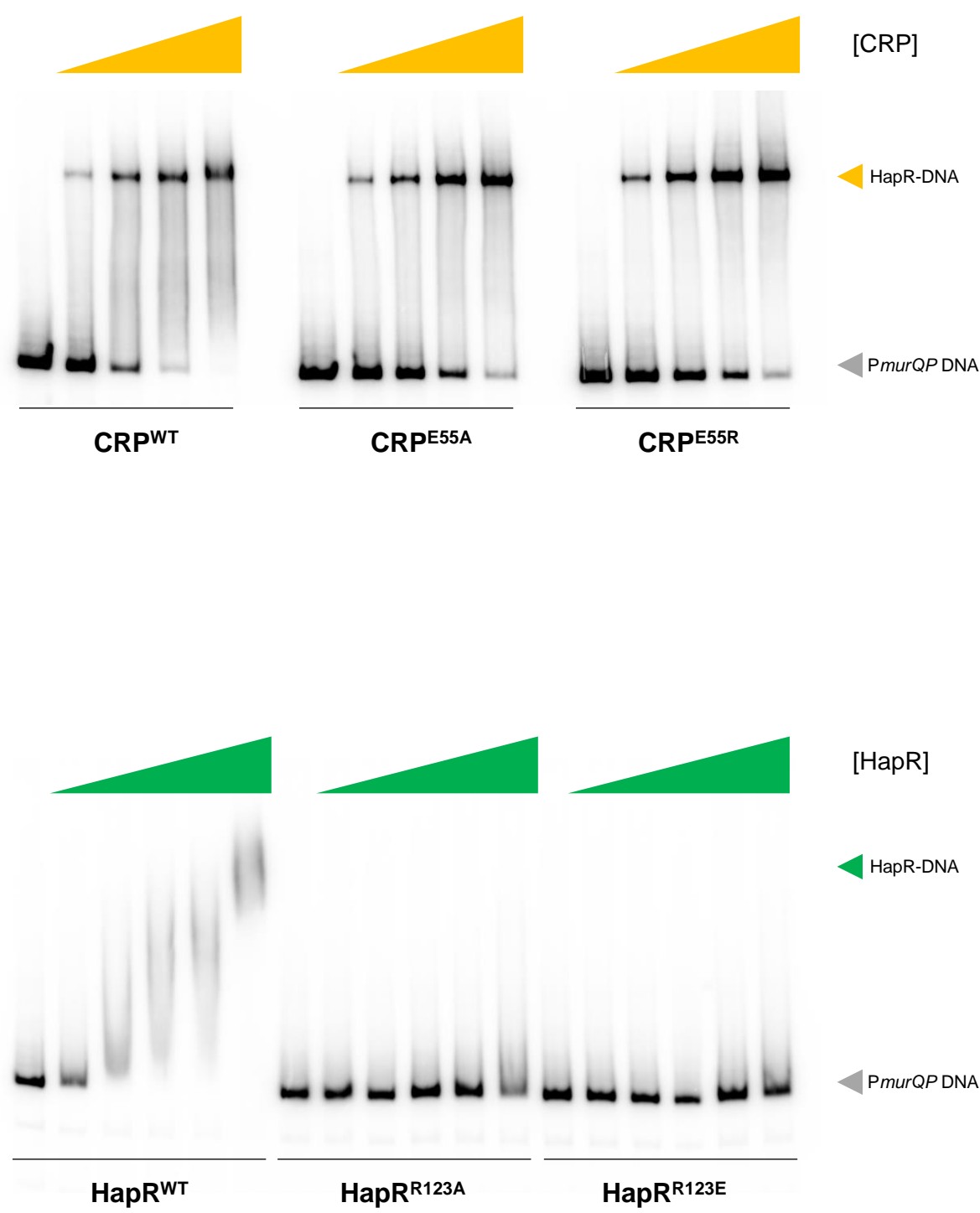

Supplement: Supplement 8 — Figure 5-figure supplement 3: Binding of CRP and HapR derivatives to PmurQP. The figures shows results of electrophoretic mobility shift assays with CRP and derivatives (0.1, 0.2, 0.4 or 0.8 μM ) or HapR and derivatives (0.25, 0.5, 1.0, 2.0 or 4.0 μM). [file media-8.pdf]

Figure 5-figure supplement 4

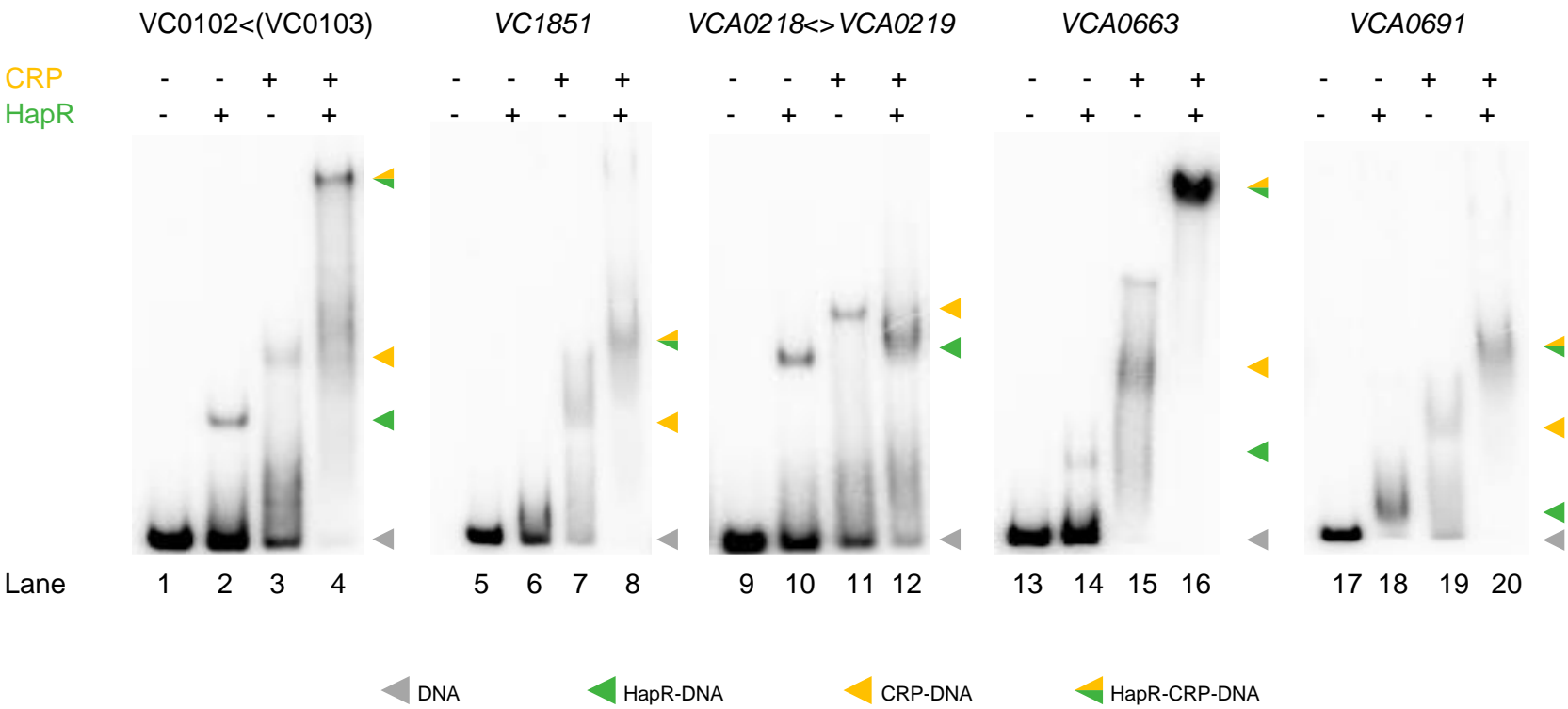

Supplement: Supplement 9 — Figure 5-figure supplement 4: Co-operative DNA binding of HapR and CRP is common. Electrophoretic mobility shift assays showing migration of the indicated regulatory regions with different combinations of CRP (1 μM) and HapR (0.19 μM). For VCA0691 the concentration of HapR was 0.57 μM. [file media-9.pdf]

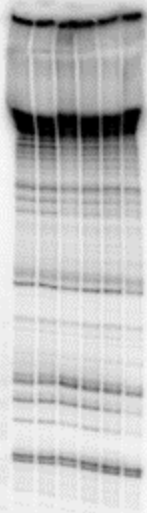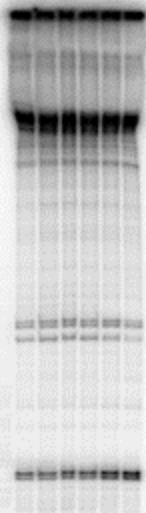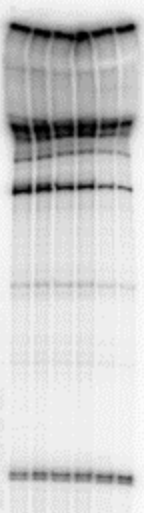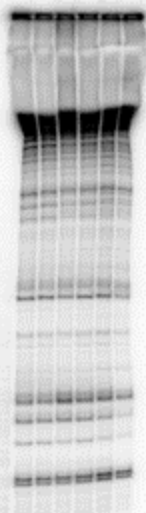

Supplement: Supplement 10 — Figure 2b source data 1: Gel image TIFF file [file media-10.pdf]

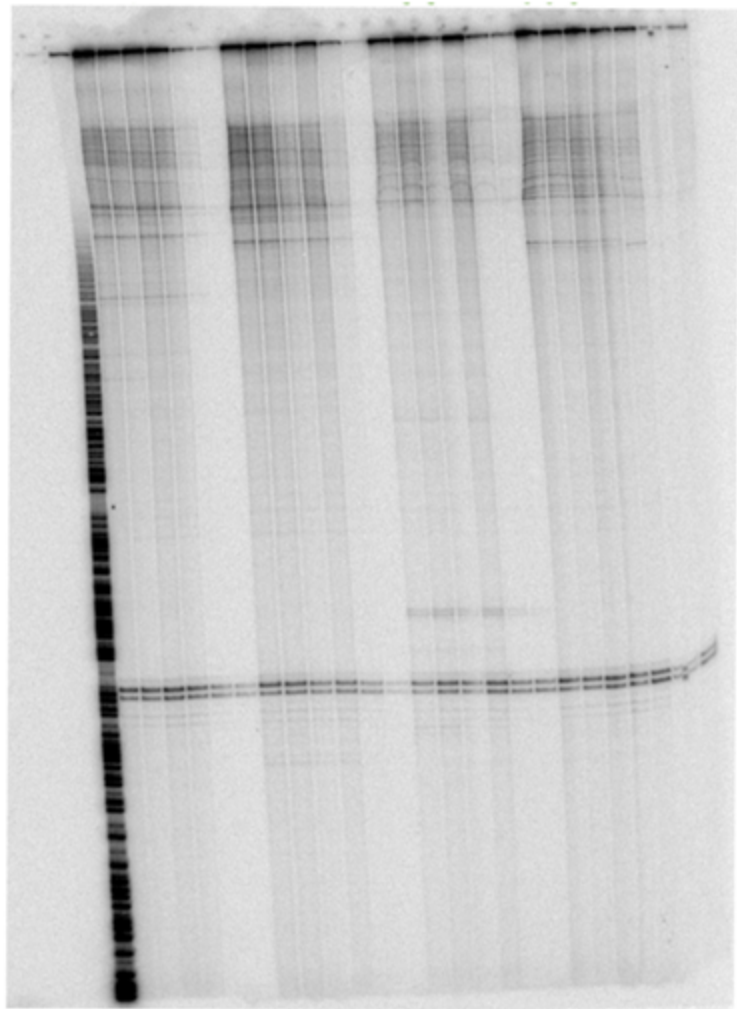

Supplement: Supplement 11 — Figure 2b source data 2: Gel image TIFF file [file media-11.pdf]

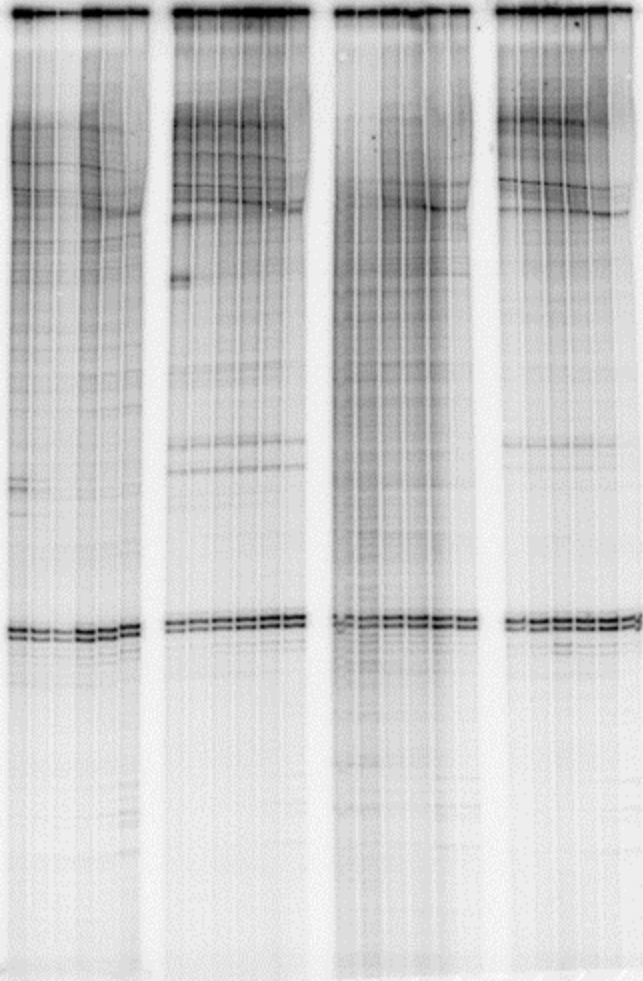

Supplement: Supplement 12 — Figure 2b source data 3: Gel image TIFF file [file media-12.pdf]

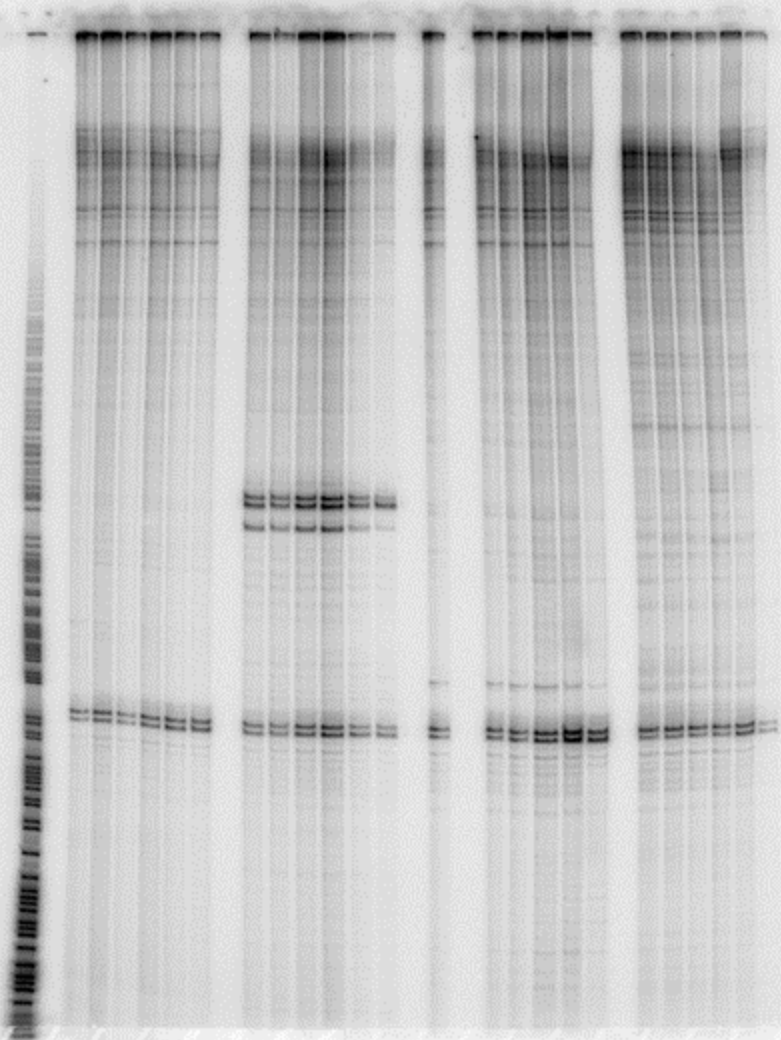

Supplement: Supplement 13 — Figure 2b source data 4: Gel image TIFF file [file media-13.pdf]

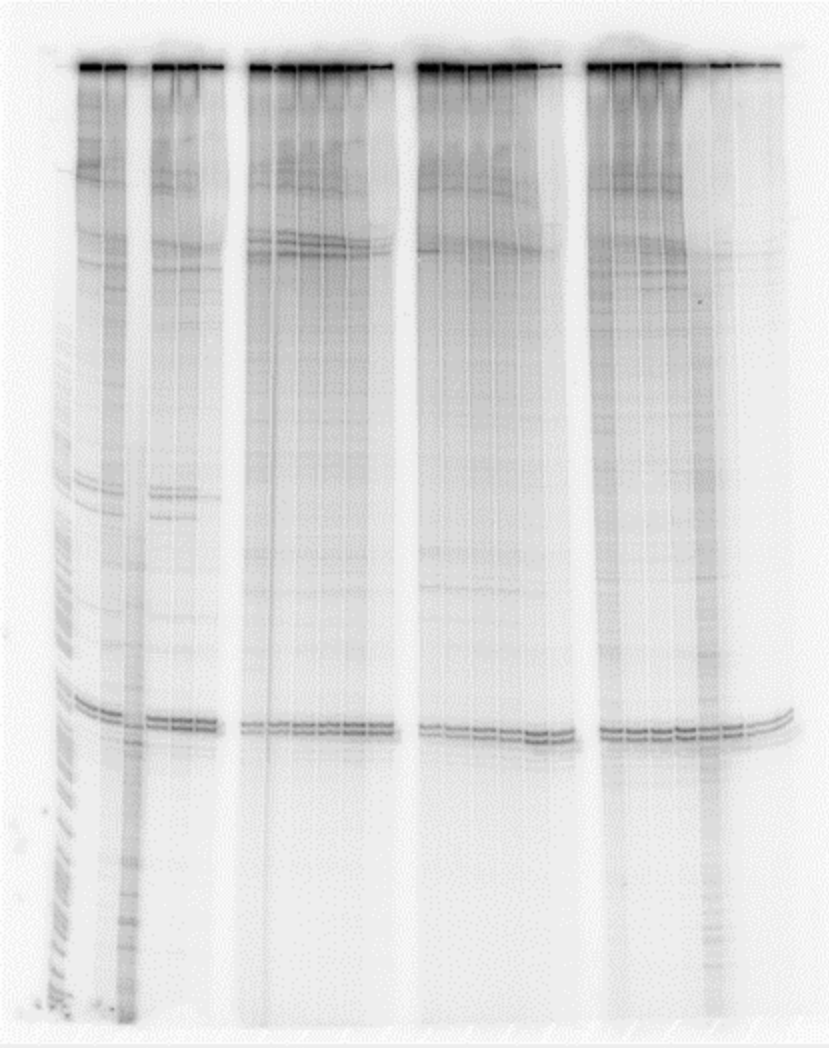

Supplement: Supplement 14 — Figure 2b source data 5: Gel image TIFF file [file media-14.pdf]

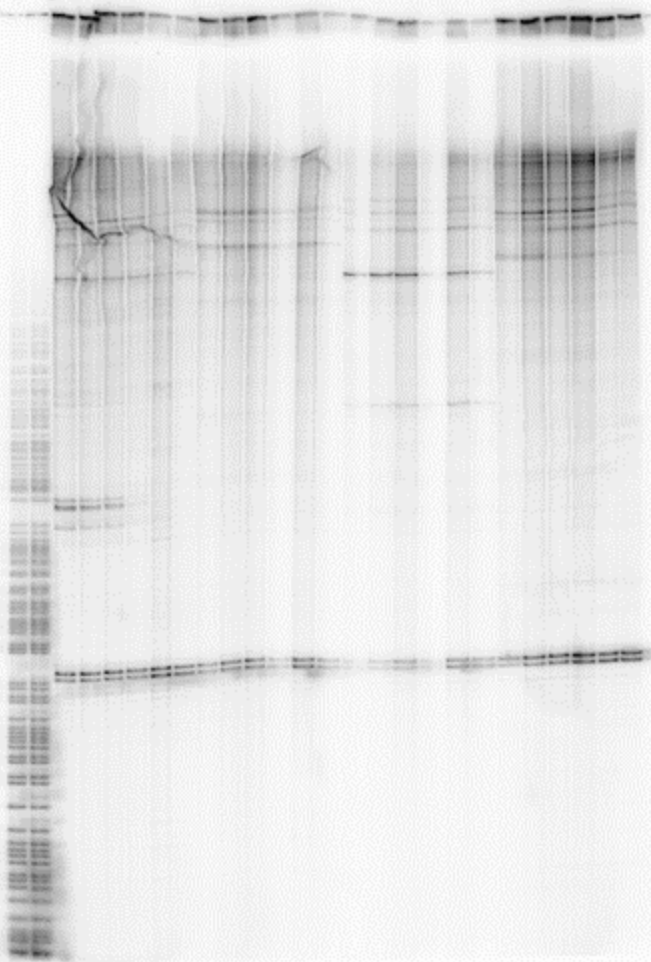

Supplement: Supplement 15 — Figure 2b source data 6: Gel image TIFF file [file media-15.pdf]

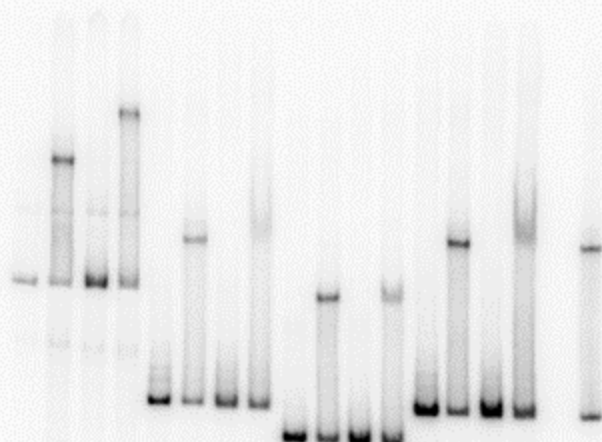

Supplement: Supplement 16 — Figure 3c source data 1: Gel image TIFF file [file media-16.pdf]

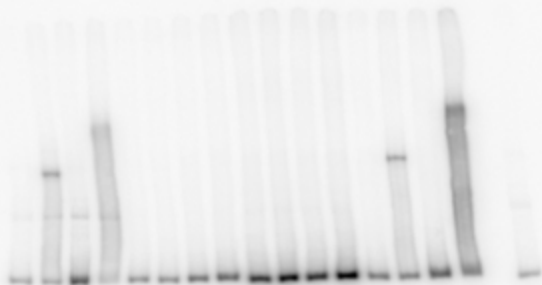

Supplement: Supplement 17 — Figure 3c source data 2: Gel image TIFF file [file media-17.pdf]

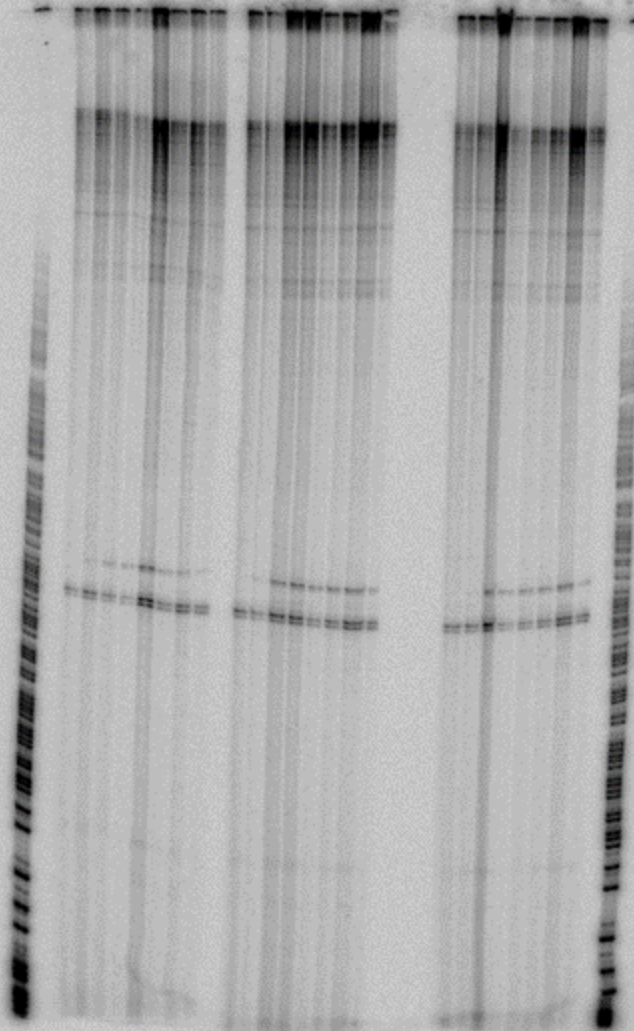

Supplement: Supplement 18 — Figure 3d source data: Gel image TIFF file [file media-18.pdf]

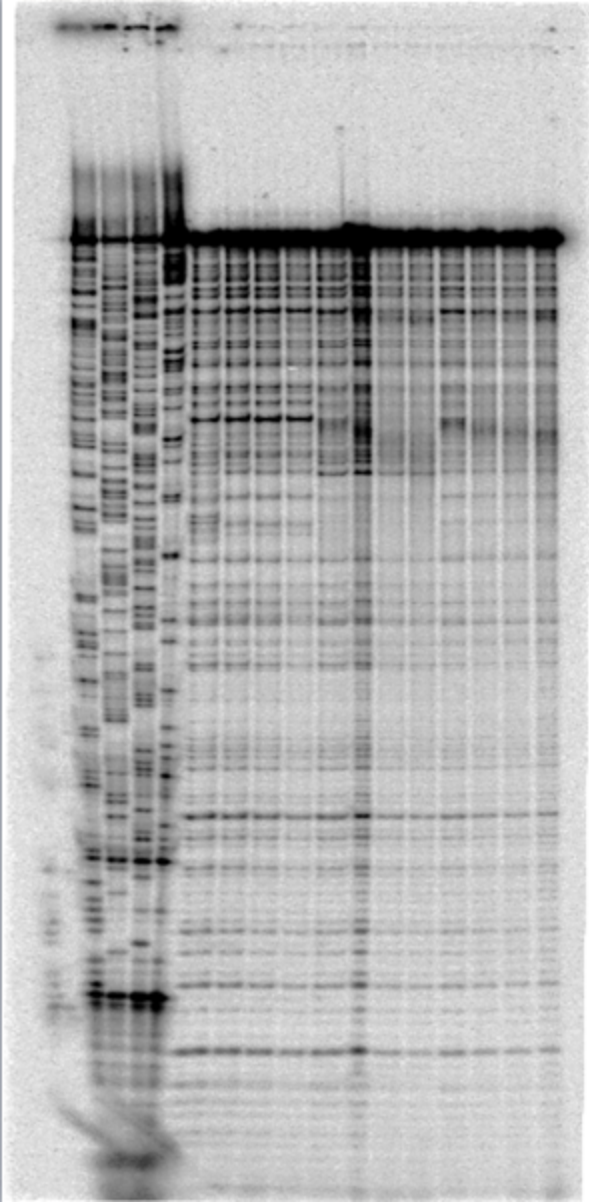

Supplement: Supplement 19 — Figure 4a source data: Gel image TIFF file [file media-19.pdf]

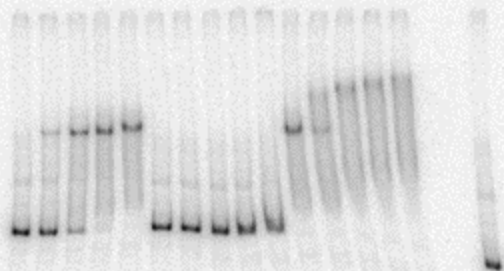

Supplement: Supplement 20 — Figure 4b source data: Gel image TIFF file [file media-20.pdf]

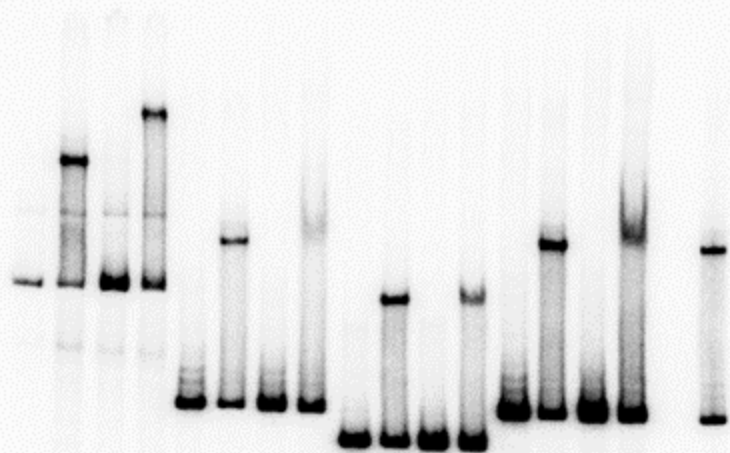

Supplement: Supplement 21 — Figure 4c source data 1: Gel image TIFF file [file media-21.pdf]

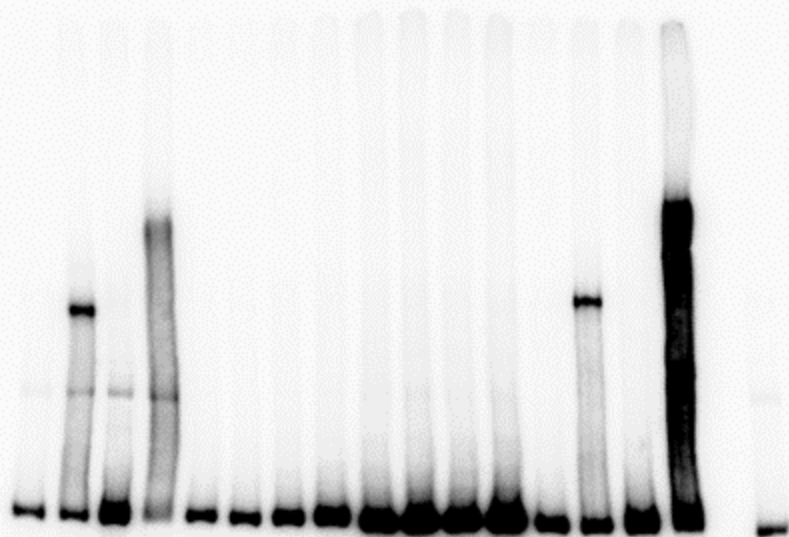

Supplement: Supplement 22 — Figure 4c source data 2: Gel image TIFF file [file media-22.pdf]

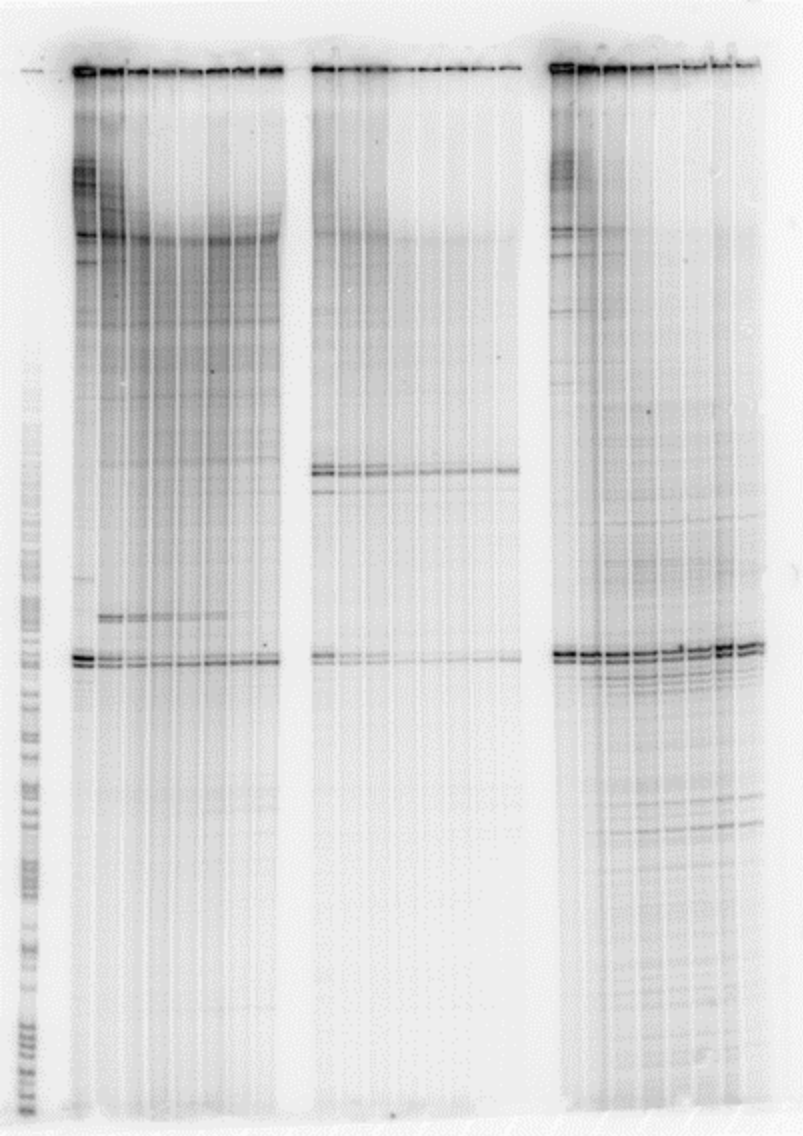

Supplement: Supplement 23 — Figure 4d source data: Gel image TIFF file [file media-23.pdf]

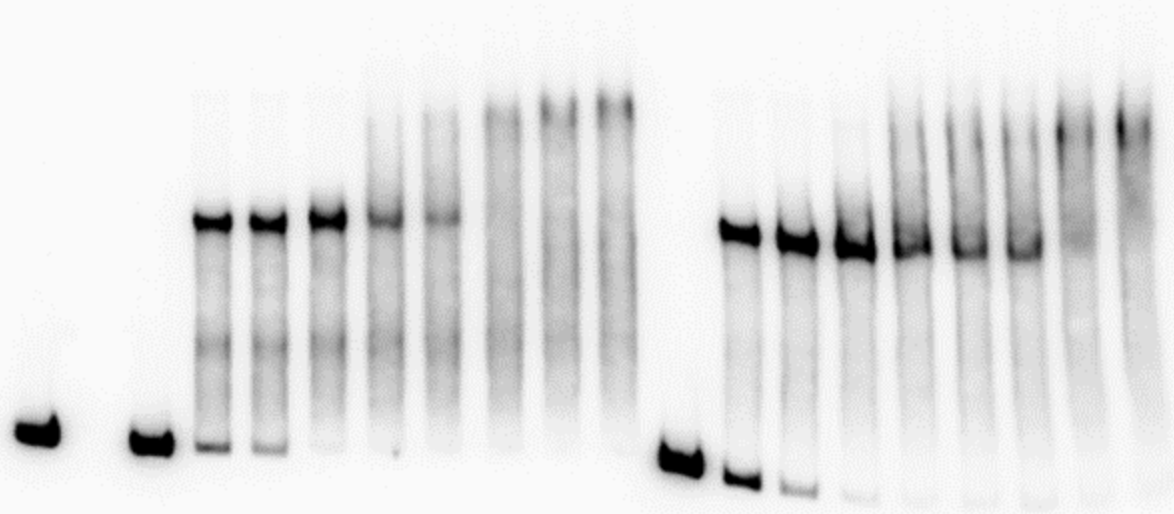

Supplement: Supplement 24 — Figure 5c source data: Gel image TIFF file [file media-24.pdf]

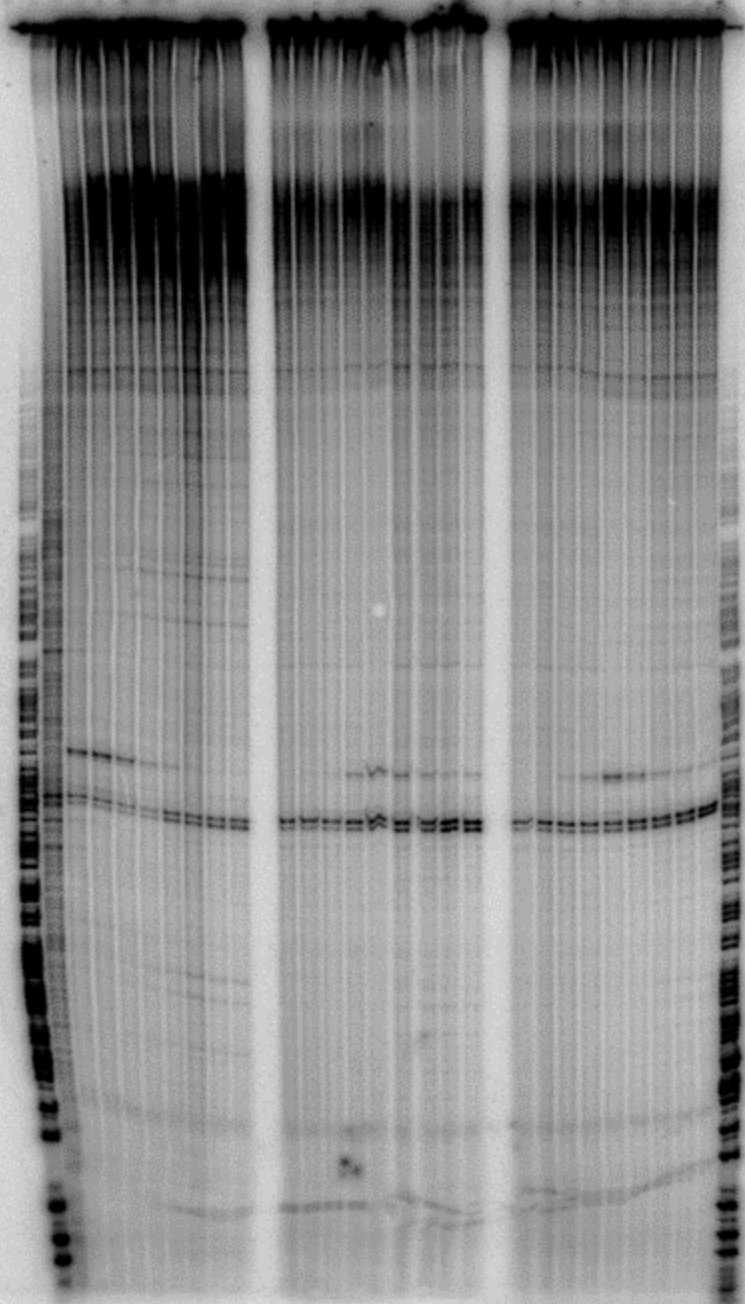

Supplement: Supplement 25 — Figure 5e source data: Gel image TIFF file [file media-25.pdf]

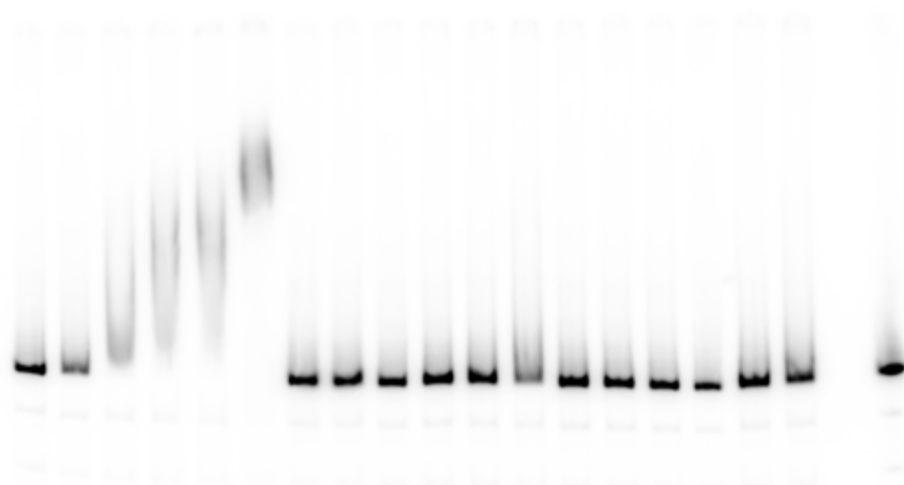

Supplement: Supplement 26 — Figure 5-figure supplement 3 source data 1: Gel image TIFF file [file media-26.pdf]

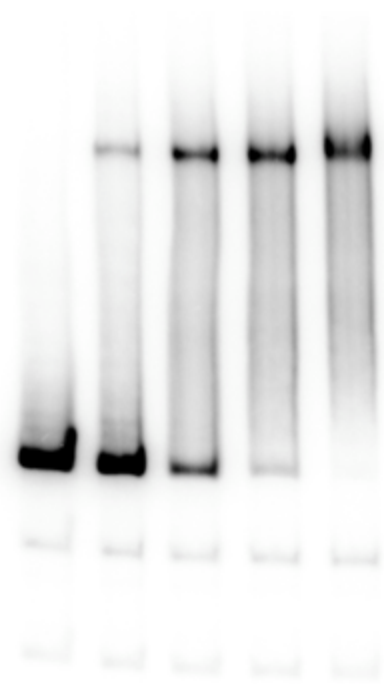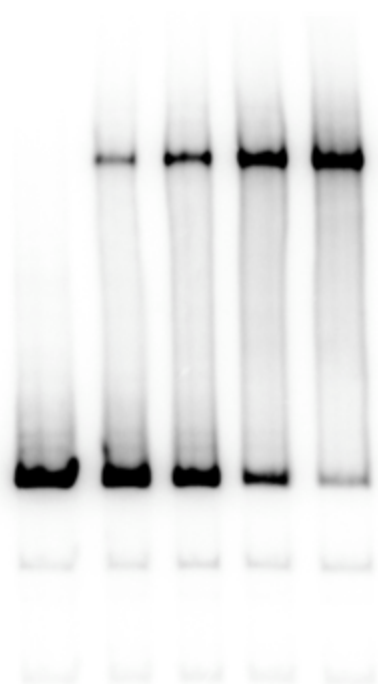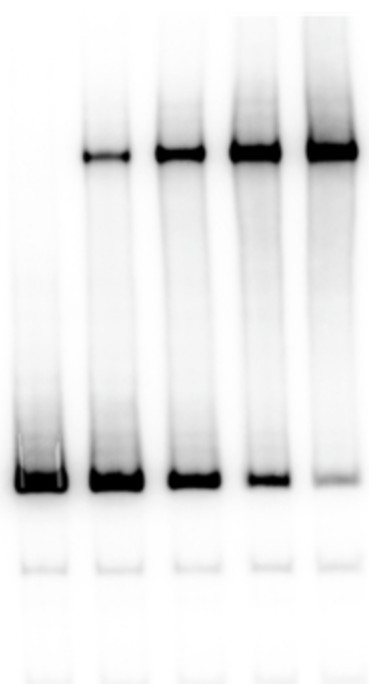

Supplement: Supplement 27 — Figure 5-figure supplement 3 source data 2: Gel image TIFF file [file media-27.pdf]

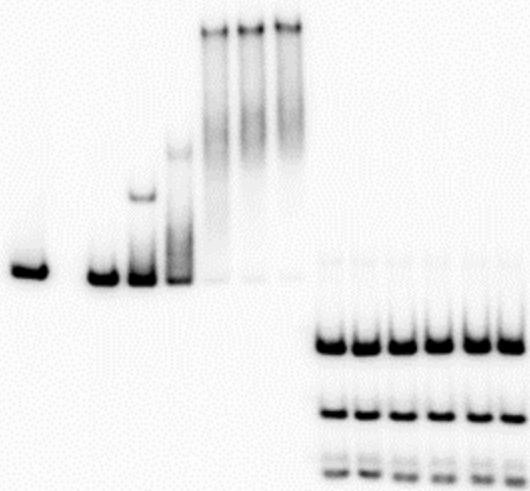

Supplement: Supplement 28 — Figure 5-figure supplement 4 source data 1: Gel image TIFF file [file media-28.pdf]

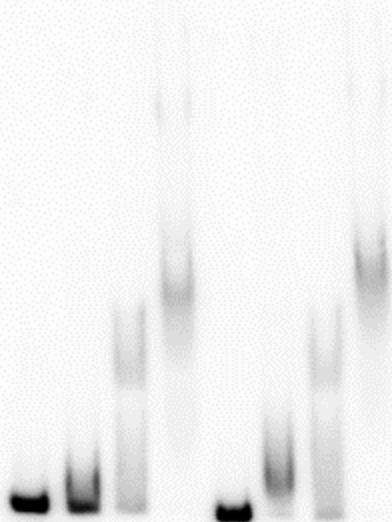

Supplement: Supplement 29 — Figure 5-figure supplement 4 source data 2: Gel image TIFF file [file media-29.pdf]

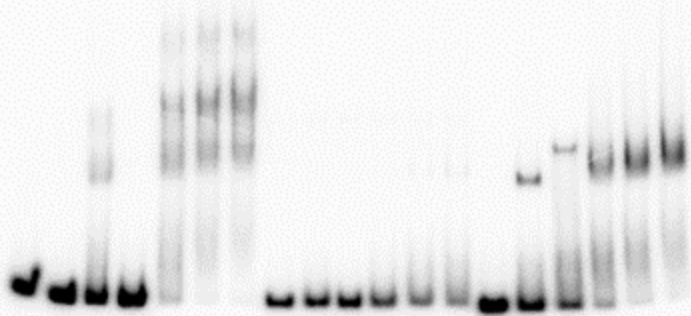

Supplement: Supplement 30 — Figure 5-figure supplement 4 source data 3: Gel image TIFF file [file media-30.pdf]

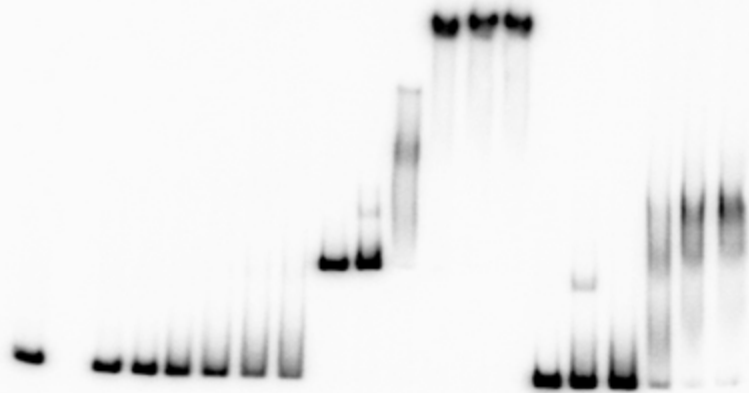

Supplement: Supplement 31 — Figure 5-figure supplement 4 source data 4: Gel image TIFF file [file media-31.pdf]
