# Supplementary material for: A simple mechanism for integration of quorum sensing and cAMP signalling in V. cholerae
Source: bioRxiv. 2023 Feb 8:2023.02.08.527633. Preprint. [Version 1] doi: 10.1101/2023.02.08.527633 (PMC9934648; doi:10.1101/2023.02.08.527633)

bioRxiv preprint doi: https://doi.org/10.1101/2023.02.08.527633; this version posted February 8, 2023. The copyright holder for this preprint (which was not certified by peer review) is the author/funder, who has granted bioRxiv a license to display the preprint in perpetuity. It is made available under aCC-BY 4.0 International license.

SUPPLEMENTARY FIGURE LEGENDS

Figure S1: Binding of LuxO and the *qrr1* and *VC1142* loci. ChIP-seq coverage plots are shown for individual experimental replicates. Signals above or below the horizontal line correspond to reads mapping to the top or bottom strand respectively.

Figure S2: Binding of HapR to the *hapR* promoter region in the presence and absence of CRP. Electrophoretic mobility shift assay showing migration of the *hapR* regulatory region with different combinations of CRP (0.0125, 0.025, 0.05 or 0.1 µM) and HapR (0.0625, 0.125, 0.25 or 0.5 µM). For incubations with both factors, the same range of HapR concentrations was used with 0.1 µM CRP.

Figure S3: Global overlap of CRP and HapR binding sites in *Vibrio harveyi*. A position weight matrix (PWM), corresponding to each DNA sequence logo shown in Figure 5a, was created. The PWMs were used to search the *V. harveyi* genome sequence (strain ATCC 33843) using FIMO. Distances between the identified CRP and HapR sites were calculated. Proximal sites were always overlapping and offset by one base pair (top panel). Overlap was greatly reduced when the analysis was applied to a randomised version of the same genome sequence (bottom panel).

bioRxiv preprint doi: https://doi.org/10.1101/2023.02.08.527633; this version posted February 8, 2023. The copyright holder for this preprint (which was not certified by peer review) is the author/funder, who has granted bioRxiv a license to display the preprint in perpetuity. It is made available under aCC-BY 4.0 International license.

Figure S4: Models of the DNA-CRP and DNA-CRP-HapR complexes. The models were generated using PDB submissions 6pb6 (*E. coli* CRP in complex with a class II CRP dependent promoter) and 1jt0 (S. aureus QacR bound to its DNA target). The DNA is shown in grey and positions hypersensitive to DNAse I cleave, in the context of the DNA-CRP complex, are highlighted red (Figure 4a). DNA position -34 is not cleaved by DNAse I in the context of the ternary DNA-CRP-HapR complex (Figure 4a). Consistent with this, position -34 is obscured by HapR binding.

Figure S5: Binding of CRP and HapR derivatives to P*murQP*. The figures shows results of electrophoretic mobility shift assays with CRP and derivatives (0.1, 0.2, 0.4 or 0.8 µM) or HapR and derivatives (0.25, 0.5, 1.0, 2.0 or 4.0 µM).

Figure S6: Co-operative DNA binding of HapR and CRP is common. Electrophoretic mobility shift assays showing migration of the indicated regulatory regions with different combinations of CRP (1 µM) and HapR (0.19 µM). For *VCA0691* the concentration of HapR was 0.57 µM.

Figure S7: Example HapR binding signals.

a. **Binding peaks for HapR that fall above our cut-off for peak selection.** The HapR ChIP-seq binding profiles are shown in green and genes are shown as blue arrows.
b. **Binding peaks for HapR, at known targets, that fall below our cut-off for peak selection.** Binding signals for HapR are shown at known target genes. These peaks for not selected by our analysis because the signal was too weak and/or insufficiently reproducible.

Figure S8: Original gel images.

Figure S1

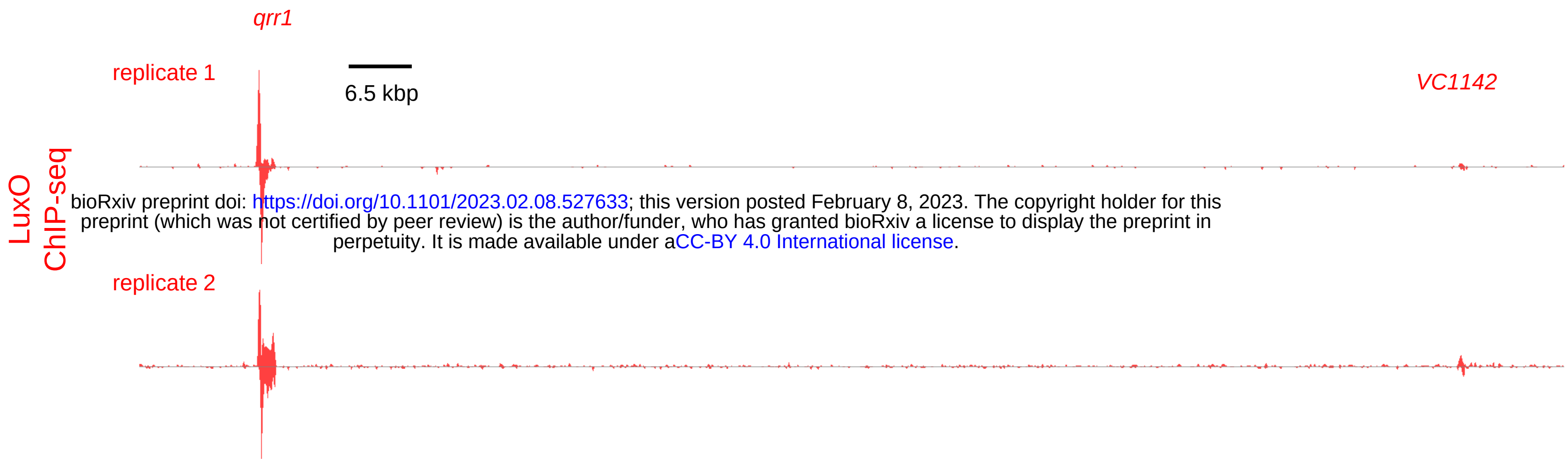

bioRxiv preprint doi: https://doi.org/10.1101/2023.02.08.527633; this version posted February 8, 2023. The copyright holder for this preprint (which was not certified by peer review) is the author/funder, who has granted bioRxiv a license to display the preprint in perpetuity. It is made available under aCC-BY 4.0 International license.

Figure S2

bioRxiv preprint doi: https://doi.org/10.1101/2023.02.08.527633; this version posted February 8, 2023. The copyright holder for this preprint (which was not certified by peer review) is the author/funder, who has granted bioRxiv a license to display the preprint in perpetuity. It is made available under aCC-BY 4.0 International license.

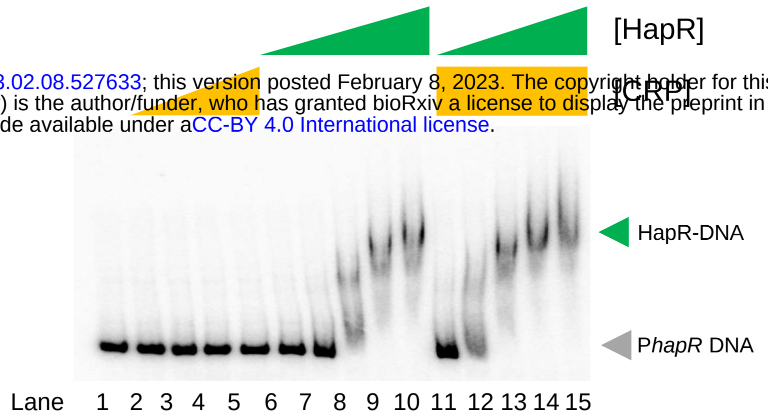

Figure S3

bioRxiv preprint doi: https://doi.org/10.1101/2023.02.08.527633; this version posted February 8, 2023. The copyright holder for this preprint (which was not certified by peer review) is the author/funder, who has granted bioRxiv a license to display the preprint in perpetuity. It is made available under aCC-BY 4.0 International license.

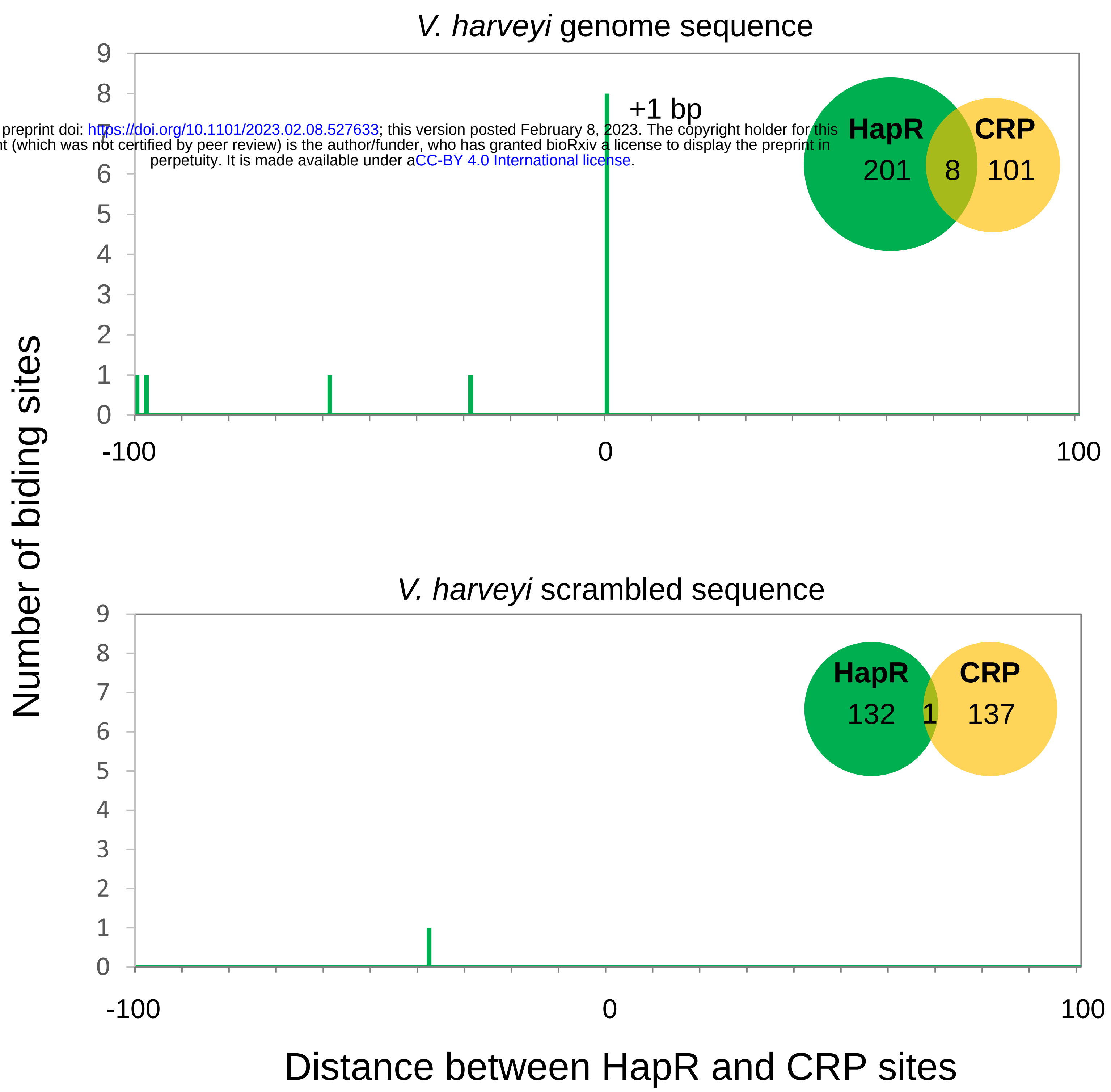

Figure S4

a

bioRxiv preprint doi: https://doi.org/10.1101/2023.02.08.527633; this version posted February 8, 2023. The copyright holder for this preprint (which was not certified by peer review) is the author/funder, who has granted bioRxiv a license to display the preprint in perpetuity. It is made available under aCC-BY 4.0 International license.

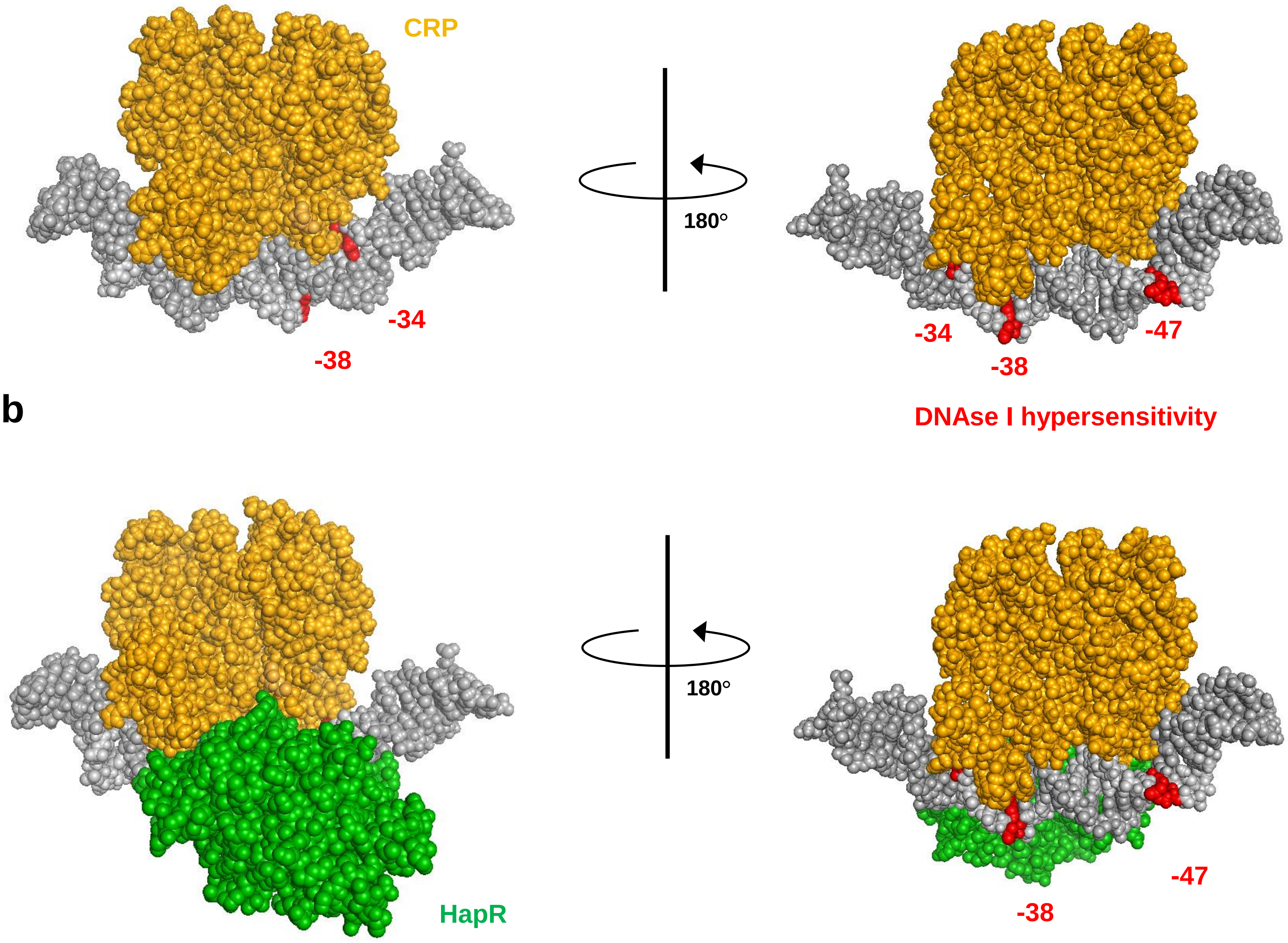

Figure S5

bioRxiv preprint doi: https://doi.org/10.1101/2023.02.08.527633; this version posted February 8, 2023. The copyright holder for this preprint (which was not certified by peer review) is the author/funder, who has granted bioRxiv a license to display the preprint in perpetuity. It is made available under aCC-BY 4.0 International license.

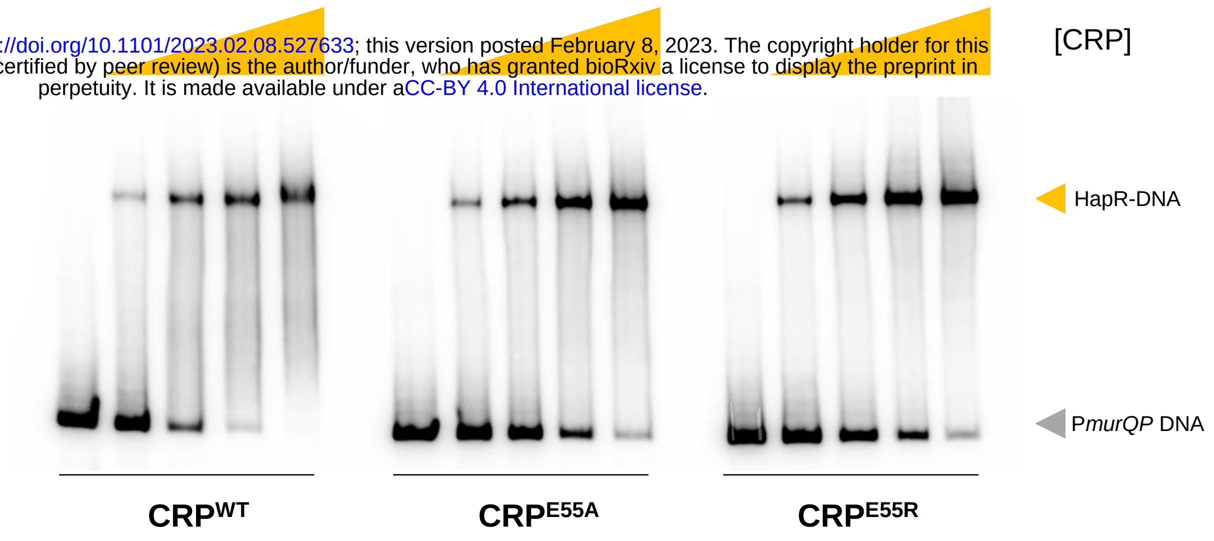

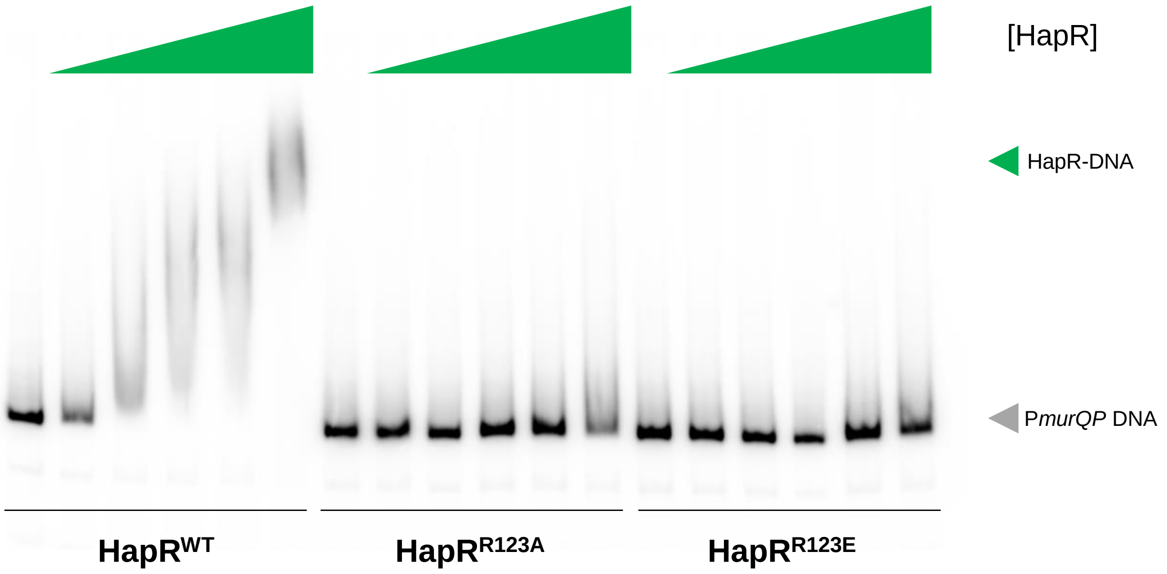

Figure S6

bioRxiv preprint doi: https://doi.org/10.1101/2023.02.08.527633; this version posted February 8, 2023. The copyright holder for this preprint (which was not certified by peer review) is the author/funder, who has granted bioRxiv a license to display the preprint in perpetuity. It is made available under aCC-BY 4.0 International license.

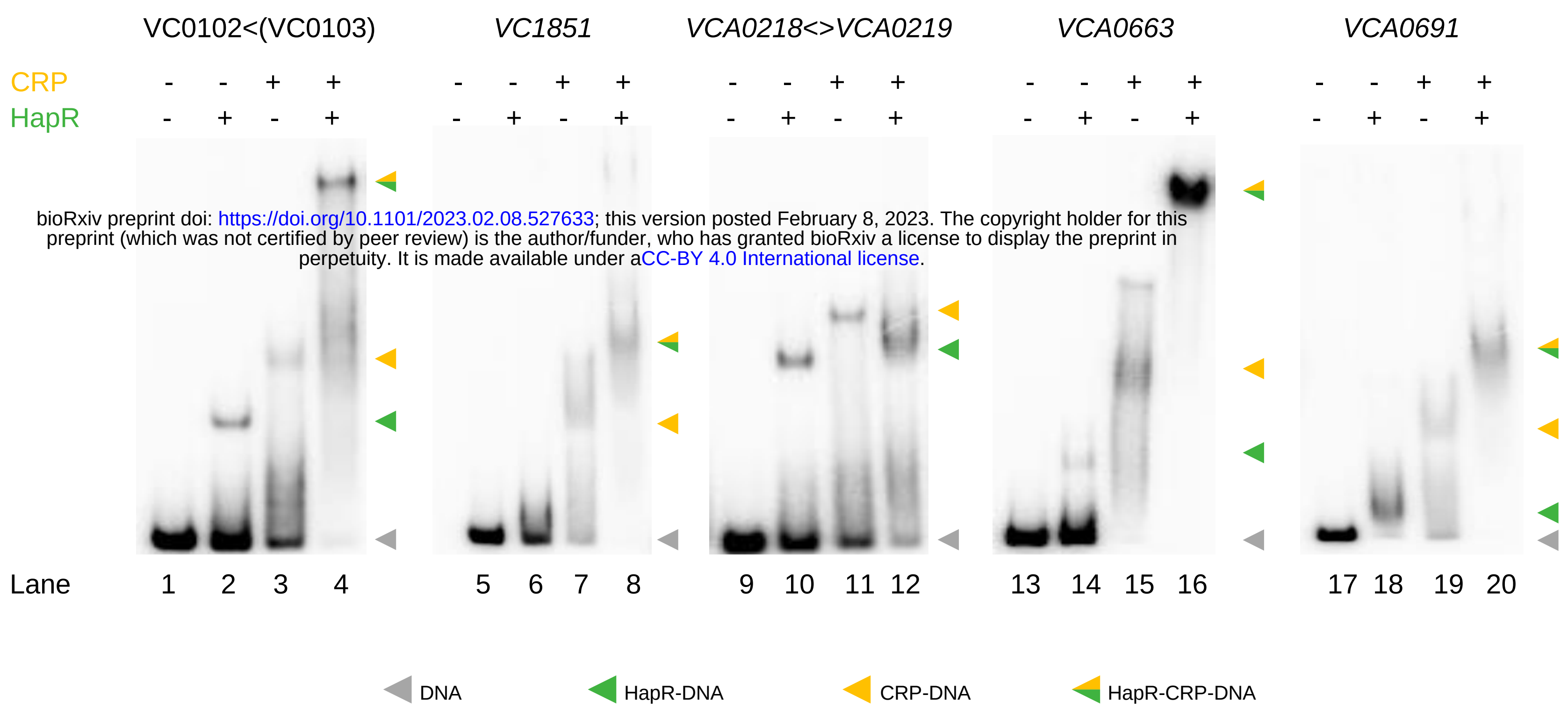

Figure S7

a

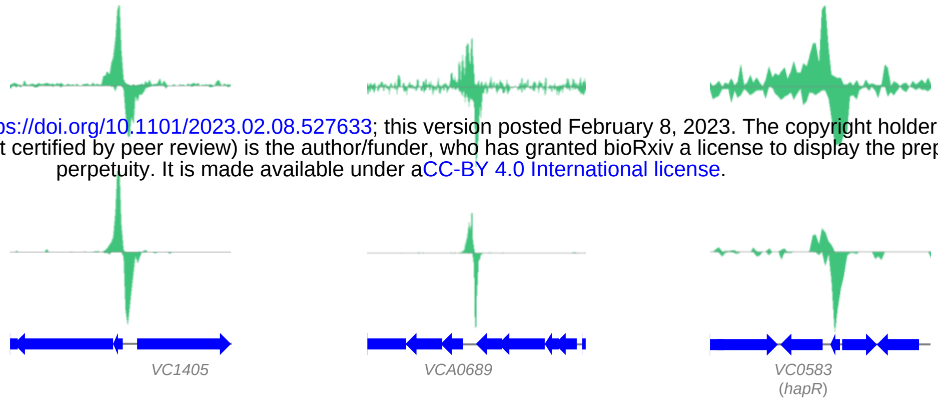

bioRxiv preprint doi: https://doi.org/10.1101/2023.02.08.527633; this version posted February 8, 2023. The copyright holder for this preprint (which was not certified by peer review) is the author/funder, who has granted bioRxiv a license to display the preprint in perpetuity. It is made available under aCC-BY 4.0 International license.

b

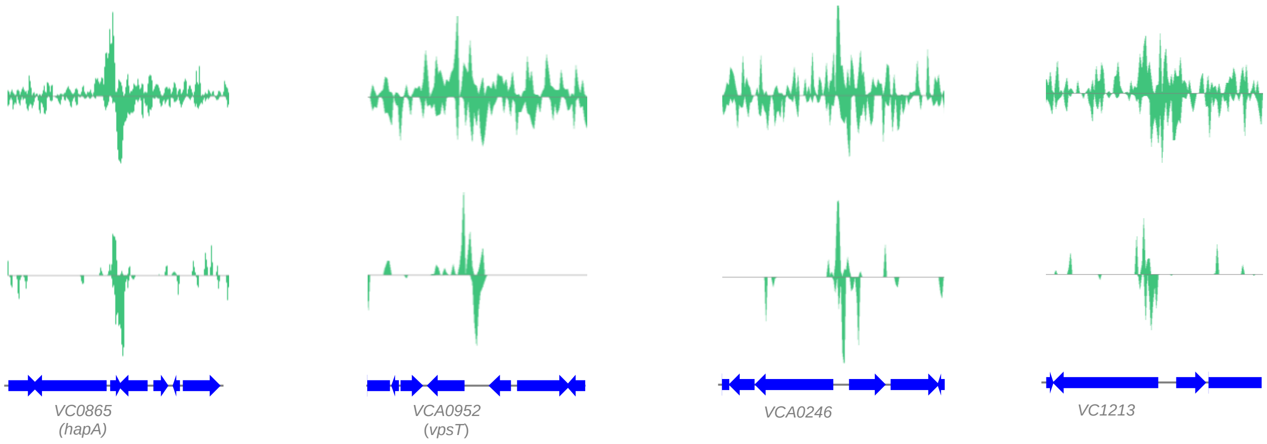

Figure S8

bioRxiv preprint doi: https://doi.org/10.1101/2023.02.08.527633; this version posted February 8, 2023. The copyright holder for this preprint (which was not certified by peer review) is the author/funder, who has granted bioRxiv a license to display the preprint in perpetuity. It is made available under aCC-BY 4.0 International license.

Figure 2b

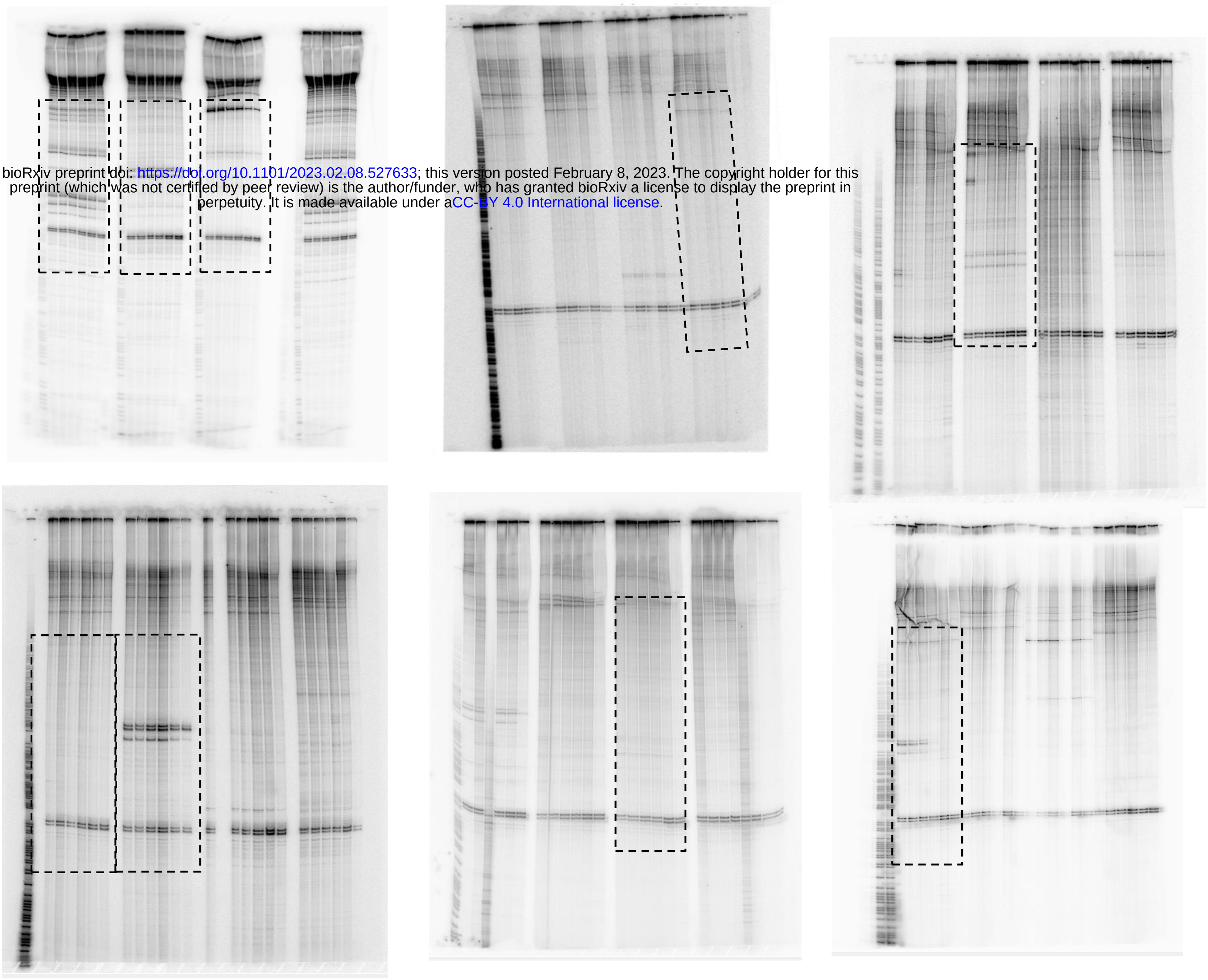

Figure 3c

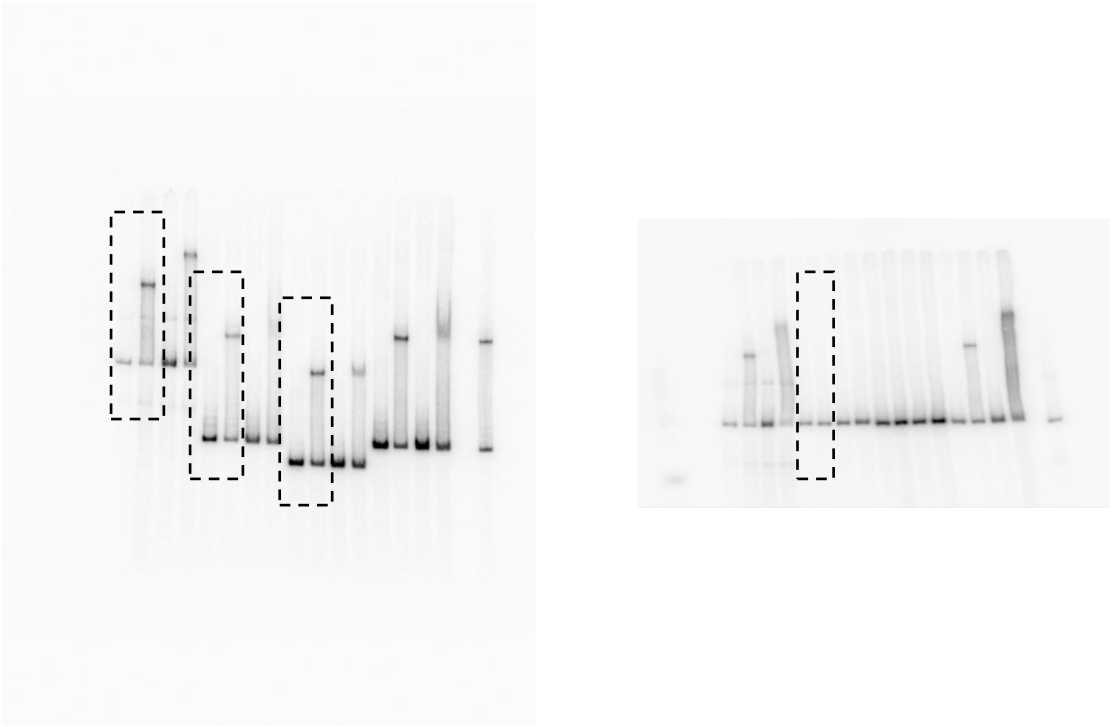

Figure 3d

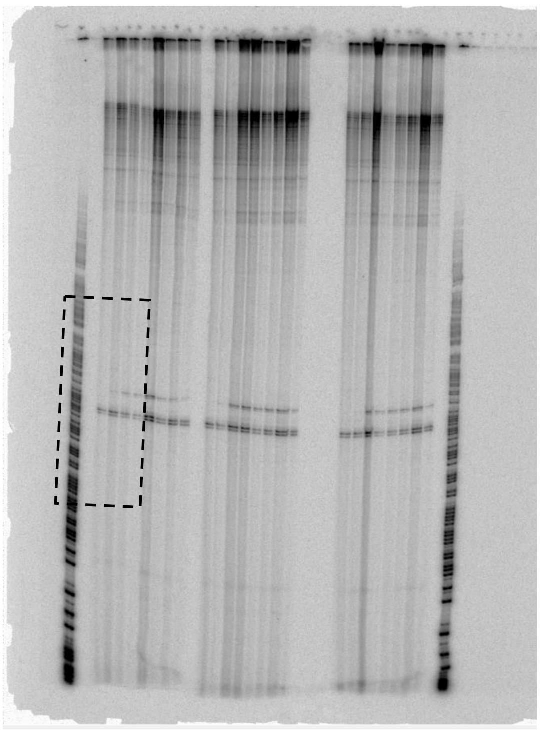

Figure 4a

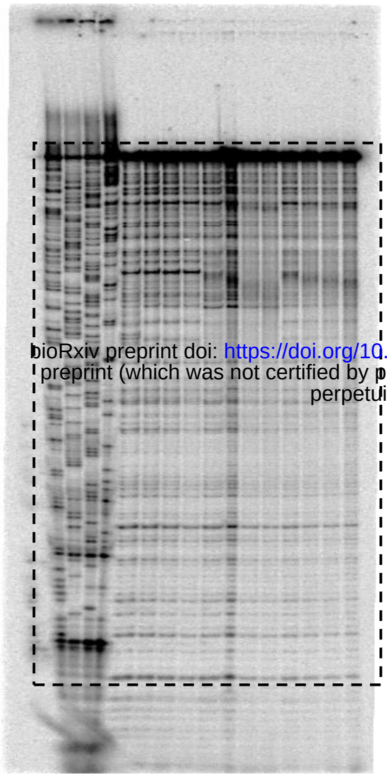

Figure 4b

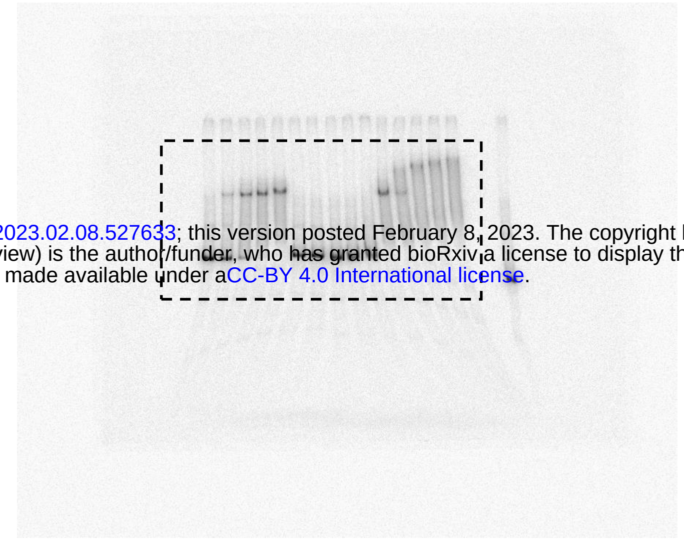

Figure 4d

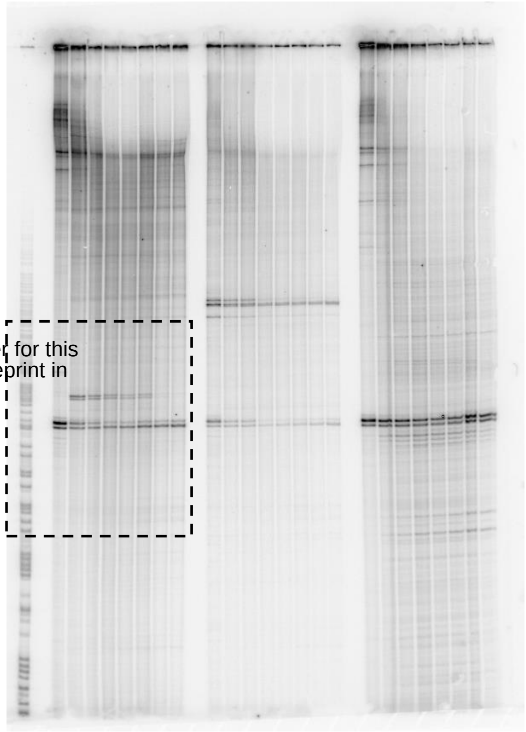

bioRxiv preprint doi: https://doi.org/10.1101/2023.02.08.527633; this version posted February 8, 2023. The copyright holder for this preprint (which was not certified by peer review) is the author/funder, who has granted bioRxiv a license to display the preprint in perpetuity. It is made available under aCC-BY 4.0 International license.

Figure 4c

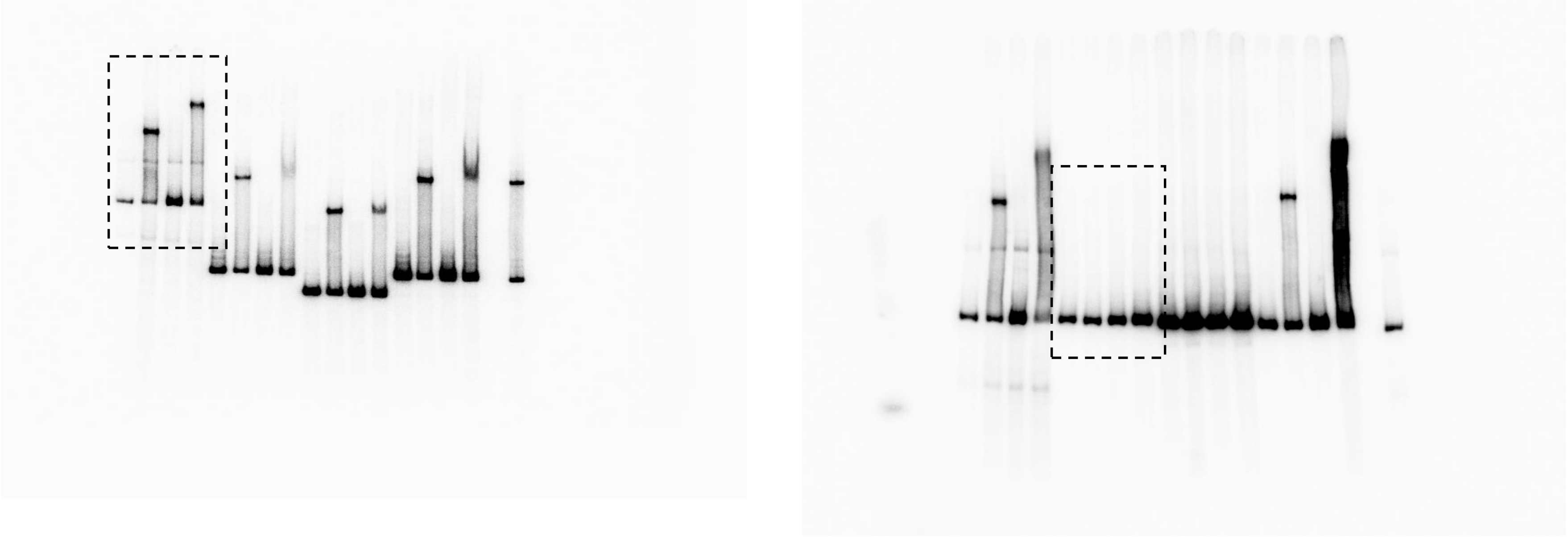

Figure 5d

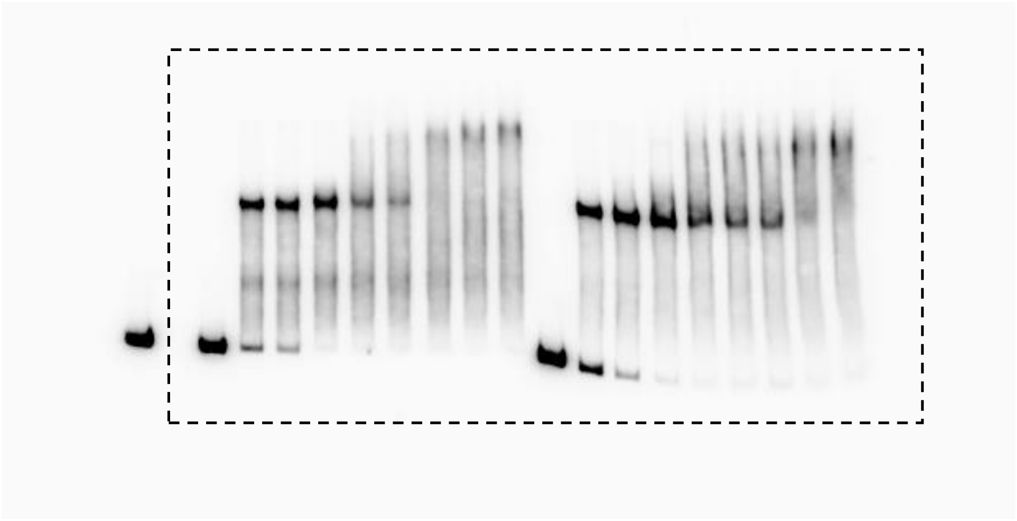

Figure 5e

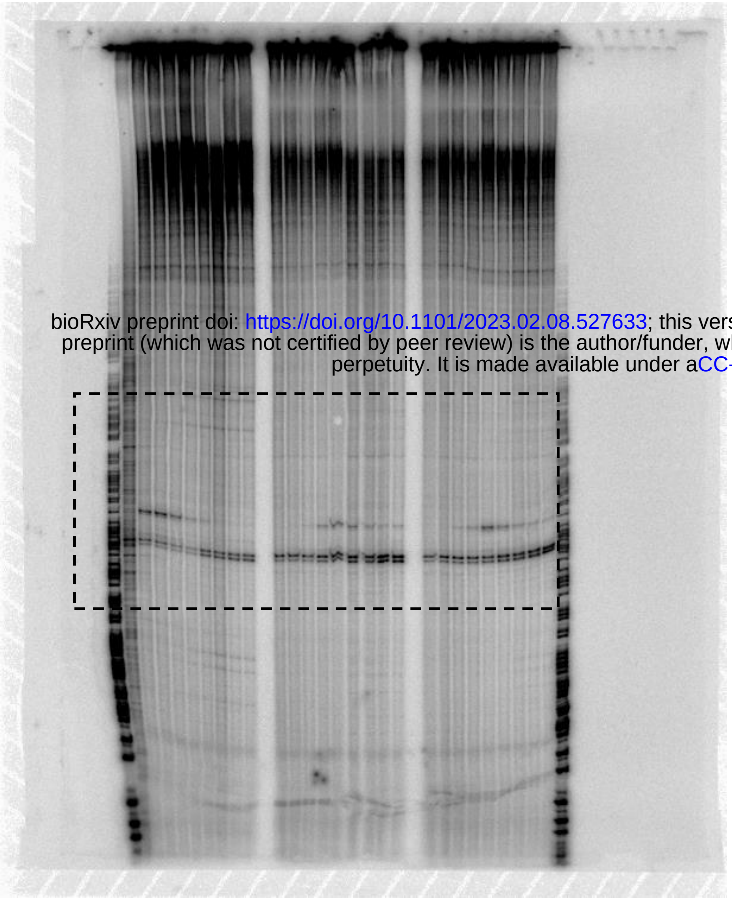

bioRxiv preprint doi: https://doi.org/10.1101/2023.02.08.527633; this version posted February 8, 2023. The copyright holder for this preprint (which was not certified by peer review) is the author/funder, who has granted bioRxiv a license to display the preprint in perpetuity. It is made available under aCC-BY 4.0 International license.

Figure S2

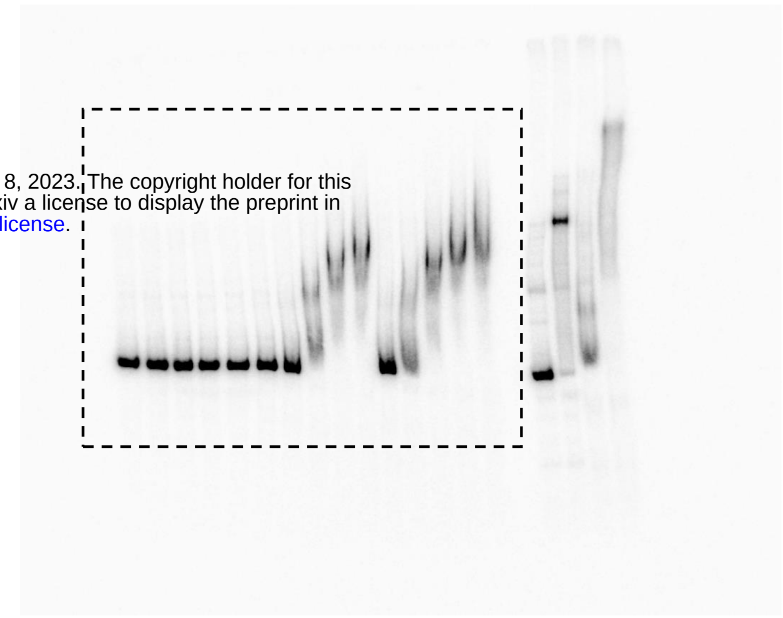

Figure S5

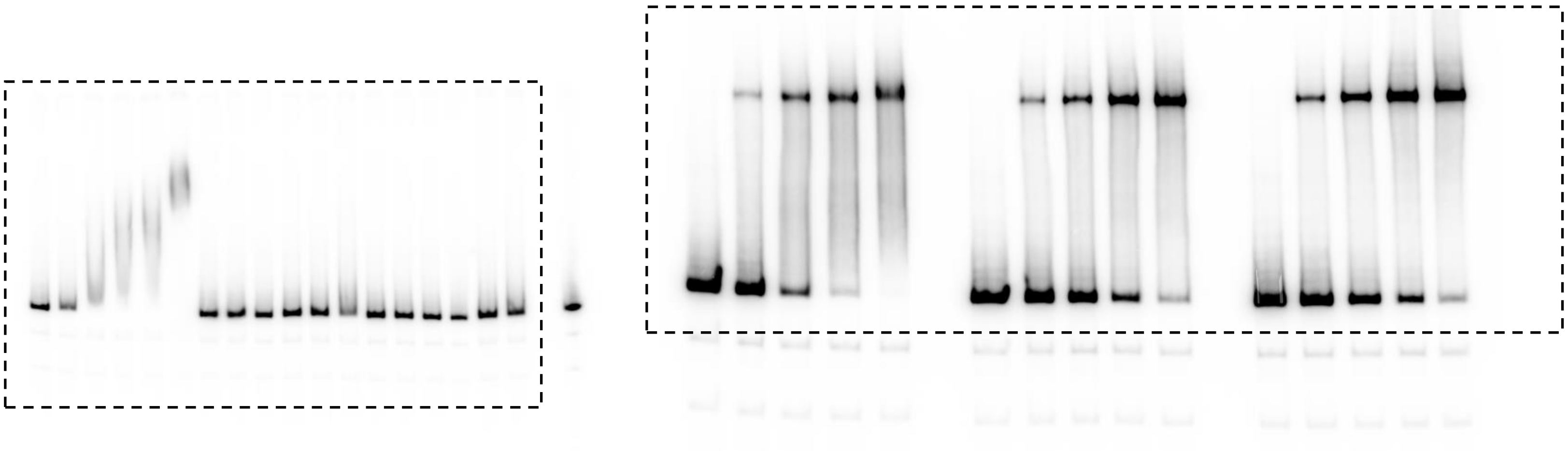

Figure S6

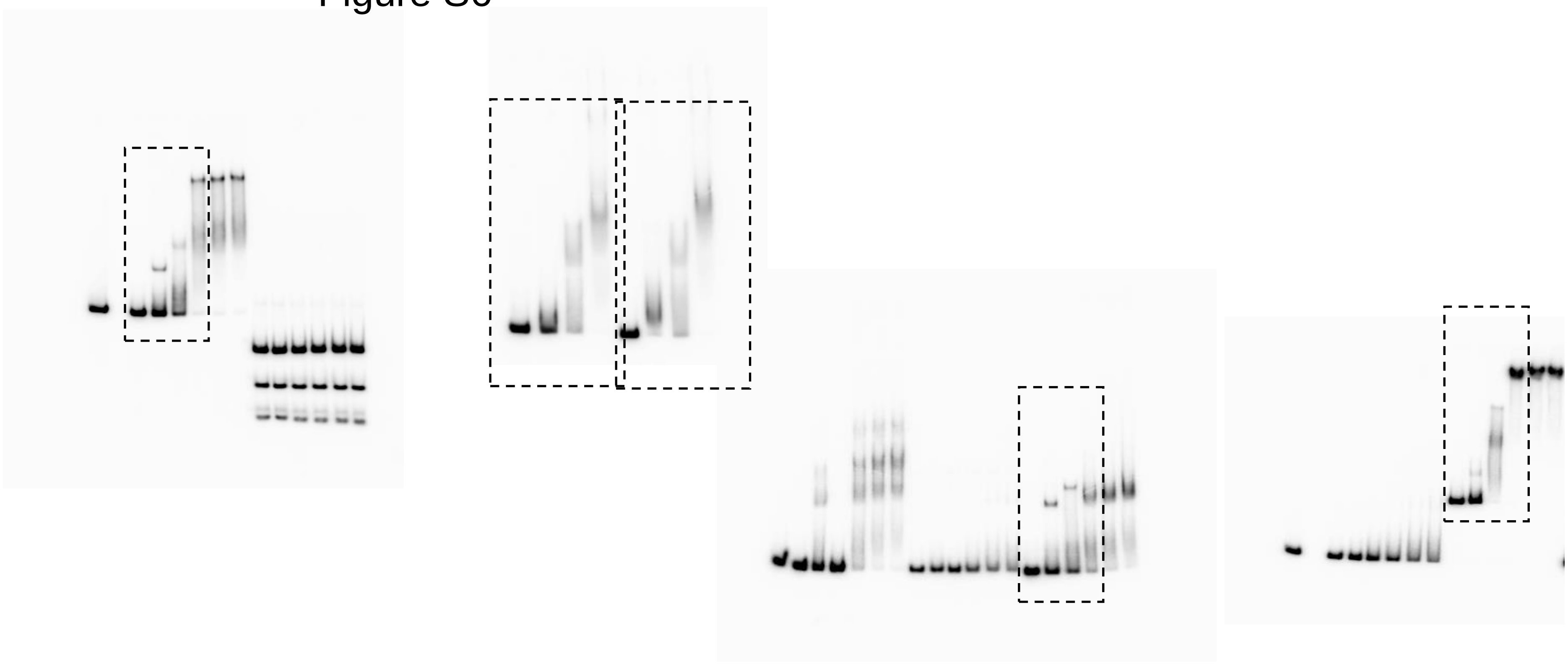

Supplement: Supplementary file 1 [file NIHPP2023.02.08.527633v1-supplement-1.pdf]
